# A multi‐omics investigation of sarcopenia and frailty: Integrating genomic, epigenomic and telomere length data

**DOI:** 10.1113/EP092853

**Published:** 2025-09-27

**Authors:** Valentina Ginevičienė, Erinija Pranckevičienė, Alina Urnikytė, Laura Jurkūnaitė, Kristijona Gutauskaitė, Rūta Dadelienė, Justina Kilaitė, Ieva Eglė Jamontaitė, Asta Mastavičiūtė, Ildus I. Ahmetov, Vidmantas Alekna

**Affiliations:** ^1^ Faculty of Medicine Vilnius University Vilnius Lithuania; ^2^ Faculty of Informatics Vytautas Magnus University Kaunas Lithuania; ^3^ Translational Health Research Institute Faculty of Medicine Vilnius University Vilnius Lithuania; ^4^ Research Institute for Sport and Exercise Sciences Liverpool John Moores University Liverpool UK

**Keywords:** ageing, epigenetics, frailty, genetics, muscle loss, sarcopenia, strength, telomere length

## Abstract

Sarcopenia and frailty are complex geriatric syndromes influenced by a combination of genetic and environmental factors. Recent studies suggest that specific genetic variants, DNA methylation patterns and shortened telomeres are associated with age‐related diseases and might contribute to the development of both sarcopenia and frailty. In this study, we investigated the contribution of multi‐omics data to sarcopenia, frailty, lean mass index (LMI) and handgrip strength in an elderly Lithuanian population. A total of 204 participants (age 82.2 ± 7.6 years) were included, comprising 122 individuals diagnosed with sarcopenia and/or frailty and 82 healthy, community‐dwelling older adults. The results showed that LMI was associated with various health and lifestyle factors. Two genetic variants, *CLIC5* rs75652203 and *GHITM* rs17102732, were found to be significantly associated with handgrip strength at the genome‐wide level. Additionally, 12 polymorphisms previously linked to sarcopenia were replicated in relationship to LMI: *BOK* rs76993203, *VAMP5* rs1374370, *TMEM18* rs12714414, *SFMBT1* rs36033494, *BANK1* rs13136118, *TET2* rs2647239, *FOXO3* rs9384679, *L3MBTL3* rs13209574, *ZFAT* rs13267329, *CEP57* rs35793328, *PCGF2* rs1985352 and *MC4R* rs66922415. Furthermore, several genes, many of which are involved in immune system processes, were significantly enriched with differentially methylated sites associated with LMI. Shorter telomeres were also associated with both sarcopenia and frailty. Notably, a significant relationship was observed between telomere length and methylation levels in genes related to lifestyle traits and the risk of developing these conditions. These findings provide new insights into the biological mechanisms underlying sarcopenia and frailty, underscoring the important roles of genetic and epigenetic factors in their pathogenesis among older adults.

## Abstract (highlights)

- **What is the central question of this study?**
  How do genetic variants, epigenetic modifications and telomere length contribute to sarcopenia and frailty‐related traits in older adults?
- **What is the main finding and its importance?**
  Two genetic variants (*CLIC5* rs75652203 and *GHITM* rs17102732) demonstrated genome‐wide significant associations with handgrip strength, and 12 single nucleotide polymorphisms were associated with lean mass index. Furthermore, differentially methylated sites in immune‐related genes were linked to lean mass index, whereas shorter telomeres were associated with sarcopenia and frailty.

## 1 INTRODUCTION

The age‐related decline in skeletal muscle mass, strength and function is associated with sarcopenia and frailty, and it increases the risk of falls, functional disability and mortality. Sarcopenia and physical frailty are progressive geriatric conditions that become increasingly prevalent with age (Cawthon et al., 2020; Ginevičienė et al., 2024). Importantly, both are complex syndromes influenced by a combination of genetic and environmental factors (Atkins et al., 2021; Mak et al., 2025; Semenova et al., 2023; Yang et al., 2025; Ye et al., 2023). Sarcopenia is defined as the loss of skeletal muscle mass, strength and function, whereas frailty is a multisystem impairment characterized by muscular atrophy, declining strength and other clinical features (da Silva et al., 2025; Ginevičienė et al., 2024). These syndromes share overlapping symptomatology, such as muscle weakness and reduced physical performance, and several common intrinsic and extrinsic predisposing factors. Consequently, there is substantial phenotypic overlap between sarcopenia and frailty, particularly in physical components such as low muscle mass, reduced grip strength and slower gait speed (da Silva et al., 2025; Ginevičienė et al., 2024).

Skeletal muscle characteristics are known to be heritable; for example, muscle strength has a heritability estimate of 49%–56% in twin and family studies (Zempo et al., 2017). However, the genetic underpinnings of this heritability, particularly in the context of age‐related syndromes, remain largely inconclusive (Jones et al., 2021). Despite extensive research, the contributors to muscle weakness in older adults are not yet fully elucidated. Recent studies suggest that genetic variants, epigenetic marks (such as DNA methylation) and shortened telomeres (protective caps at chromosome ends) are associated with ageing and age‐related diseases. These factors might contribute to the development of both sarcopenia and frailty by accelerating cellular ageing and reducing the capacity for tissue repair and regeneration, including in skeletal muscle (Kmiołek et al., 2023). Genome‐wide association studies (GWAS) have identified several common single nucleotide polymorphisms (SNPs) associated with sarcopenia‐ or frailty‐related phenotypes, including strength (Jones et al., 2021; Moreland et al., 2022; Tikkanen et al., 2018), lean mass (Pei et al., 2020), muscle fibre size (Guilherme et al., 2022, 2024), power performance (Maciejewska‐Skrendo et al., 2019) and walking speed (Timmins et al., 2020).

Epigenetic mechanisms, such as DNA methylation, are also known to modulate molecular pathways of ageing. DNA methylation is catalysed by DNA methyltransferases, which attach a methyl group to the 5′ carbon of cytosine residues in CpG sites (regions of DNA where a cytosine is followed by a guanine). Methylation of promoter regions is typically correlated with gene expression levels: hypermethylation is often associated with gene silencing, whereas hypomethylation can activate gene expression. This regulation occurs in a tissue‐specific manner. Telomeres represent another key molecular feature linked to genome stability, ageing and age‐related diseases. These dynamic structures protect chromosomes from degradation and instability. Age‐related telomere shortening results from interactions between genetic and environmental factors and is considered a potential biomarker for sarcopenia and frailty. Nonetheless, the roles of telomere length and DNA methylation in muscle ageing and the pathogenesis of sarcopenia and frailty remain poorly understood (Kmiołek et al., 2023).

Timely identification of predictors for adverse body composition phenotypes in geriatric syndromes, using molecular markers, might facilitate the development of targeted preventive strategies, an essential step towards precision medicine. The aim of the present study was to identify intrinsic factors significantly associated with the lean mass index (LMI), a height‐adjusted measure used as a key diagnostic criterion for sarcopenia and frailty. In this study, we investigated the contribution of multi‐omics data to sarcopenia, frailty, LMI and handgrip strength in an elderly Lithuanian population. It was hypothesized that telomere length, genomic variation and epigenomic markers might be associated with interindividual variability in LMI and handgrip strength.

## 2 MATERIALS AND METHODS

### 2.1 Ethical approval

The study conformed to the standards set by the *Declaration of Helsinki*, except for registration in a database, and received approval from the Lithuanian Regional Biomedical Research Ethics Committee (no. 2022/6‐1448‐918). All participants provided written informed consent prior to enrolment.

### 2.2 Participants

This cross‐sectional study included community‐dwelling adults aged ≥65 years, recruited through older adult societies and a geriatric centre. Inclusion criteria were age ≥65 years and community residence. Exclusion criteria included impaired basic daily function, active cancer treatment, acute illness, chronic renal failure, rheumatoid arthritis, heart disease or moderate cognitive impairment (Mini‐Mental State Examination score <21). A total of 204 participants (43 men and 161 women; mean age 82.2 ± 7.6 years) were enrolled, of whom 181 were genotyped successfully. The control group included 82 healthy individuals (17 men and 65 women; 80.2 ± 7.5 years), whereas 122 participants (26 men and 95 women; 85.3 ± 6.7 years) had sarcopenia and/or frailty: 31 had sarcopenia only, 29 had frailty only, and 62 had both. Sarcopenia was diagnosed according to European Working Group on Sarcopenia in Older People 2 guidelines (EWGSOP2) criteria (Cruz‐Jentoft et al., 2019), requiring low muscle strength and mass, and was classified as severe if low physical performance was also present. Frailty was defined using Fried's criteria (Fried et al., 2001), with diagnosis based on at least three of five components: weight loss, weakness, exhaustion, slowness and low activity.

### 2.3 Phenotype assessment

Participants completed a questionnaire covering socioeconomic factors (education level, marital status, housing and income), smoking and alcohol consumption. Information on the number of diseases and medications taken was obtained from medical records. Polypharmacy was defined as the use of five or more medications. Nutritional status was assessed using the Mini Nutritional Assessment–Short Form (Rubenstein et al., 2001). Depressive symptoms were screened using the 15‐item Geriatric Depression Scale (GDS; https://geriatrictoolkit.missouri.edu/cog/GDS_SHORT_FORM.PDF; accessed 5 April 2025).

Physical performance was measured using the Short Physical Performance Battery (SPPB; https://geriatrictoolkit.missouri.edu/SPPB‐Score‐Tool.pdf; accessed 5 April 2025). Physical activity was assessed with the Physical Activity Scale for the Elderly (PASE). Everyday functioning was evaluated using two questionnaires: Activities of Daily Living (ADL) and Instrumental Activities of Daily Living (IADL).

All participants were measured for height (using a stadiometer, accurate to the nearest 0.1 cm) and weight (using an electronic scale, accurate to the nearest 0.1 kg). Body mass index (in kilograms per metre squared) was calculated from these measurements. Body composition (lean mass, fat mass and fat mass percentage) was assessed by dual‐energy X‐ray absorptiometry (iDXA, GE Lunar, USA). The LMI was calculated by dividing lean mass by height squared. Muscle strength was evaluated using handgrip strength, measured with a hydraulic dynamometer (JAMAR, Patterson Medical, UK), following the Southampton protocol (Roberts et al., 2011). Both the DXA machine and the dynamometer were calibrated according to the manufacturers’ instructions.

Physical performance was assessed further with a 4 m walking test. The cut‐off values recommended by the EWGSOP consensus were applied. A gait speed of <0.8 m/s was considered indicative of low physical performance. Low muscle strength was defined as handgrip strength <27 kg for men and <16 kg for women. Low muscle mass was defined as an LMI of <8.87 kg/m^2^ for men and <6.42 kg/m^2^ for women (Cruz‐Jentoft et al., 2019).

Psychomotor speed was assessed through reaction time and movement frequency using a tapping test. A personal computer, a specialized software program and a reactiometer RA‐1 (JSC Baltec CNC Technologies, Lithuania) were used. The device was calibrated according to the manufacturer's instructions. Simple reaction time was assessed by having participants press a button with their right hand as quickly as possible in response to a light signal. Complex reaction time was measured by instructing participants to press the button with the right hand when the green light appeared and with the left hand when the red light appeared. Errors were recorded if the incorrect button was pressed. The tapping test was conducted by having participants hold a stick and tap a board as rapidly as possible after an auditory signal. Tapping frequency was recorded over 10 and 60 s intervals.

Participants underwent a 6 min cycle ergometer test (ERGO‐FIT 777, Germany) with a workload of 40 W at 50 rpm. Heart rate and blood pressure were measured at the end of the test. Heart rate was recorded every minute for 5 min postexercise, and blood pressure was measured 5 min after completion. The modified Borg scale was used to assess perceived exertion, with higher scores indicating greater effort.

Muscle oxygenation was assessed at rest, during exercise and throughout passive recovery using near‐infrared spectroscopy. The sensor was placed on the vastus lateralis muscle, 10 cm above the proximal border of the patella. After a warm‐up, participants performed a 6 min aerobic exercise on the ergometer at 60 W and 60 rpm. Muscle oxygen saturation [$S_{mO_{2}}$ (%)] and total haemoglobin concentration (in grams per decilitre) were measured at baseline (after 5 min of rest in a sitting position), at the end of the exercise and at each minute during a 5 min passive recovery period. Heart rate responses were monitored telemetrically.

### 2.4 Genotyping

Genomic DNA for multi‐omic analyses (including GWAS, methylation profiling and measurement of relative telomere length) was extracted from peripheral venous blood using the PureLink Genomic DNA Mini Kit (Thermo Fisher Scientific, USA) following the manufacturer's instructions. Blood was selected as the DNA source owing to its accessibility, well‐established processing protocols and suitability for large‐scale population‐based studies. Although venipuncture is a routine clinical procedure, it is considered minimally invasive in comparison to tissue biopsy methods.

DNA concentration and purity were assessed using a NanoDrop ND‐1000 spectrophotometer (Thermo Fisher Scientific, USA). Genotyping was carried out using the Illumina Infinium Global Screening Array‐24 v.3.0 Kit (Illumina, San Diego, CA, USA), which includes 654 027 genome‐wide SNPs. Quality control of the genotyping data was performed in accordance with standard manufacturer's recommendations. Genotype data were filtered based on the following criteria: SNP genotype missingness of >10%, sample missingness of >10%, SNP minor allele frequency of <1%, and deviation from Hardy–Weinberg equilibrium (*p* < 10^−4^). Of the initial 654 027 SNPs, 10 549 were excluded owing to missing genotype data, and 145 variants were removed based on the Hardy–Weinberg equilibrium test. Individuals with a call rate of <0.9 or heterozygosity of >0.4 were also excluded. To identify population outliers, multidimensional scaling analysis was performed using independent SNPs in PLINK v1.07. SNP pruning was conducted in PLINK (https://doi.org/10.1086/519795; accessed 5 April 2025), with a window size of 50 SNPs, a step size of five SNPs, and an *r* ^2^ threshold of 0.5. Pairwise relatedness was evaluated using pi‐hat statistics, and kinship analysis was conducted using KING v.2.3.2 (https://doi.org/10.1093/bioinformatics/btq559; accessed 5 April 2025). Samples identified as outliers in the multidimensional scaling plot, in addition to individuals with pi‐hat of >0.2 or a kinship coefficient of >0.0084, were excluded from further analysis.

Before genotype imputation, an initial association analysis was performed using PLINK (v.1.07 and v.1.9). Age and sex were included as covariates in logistic and linear regression models to control for variation in age and sex distribution among participants. Empirical *p*‐values were obtained through permutation testing with 10 000 permutations to adjust for multiple testing. Genotyping data were phased using EAGLE v.2.4.1 (Loh et al., 2016). Imputation was performed with BEAGLE v.5.5 (Browning et al., 2021) using the 1000 Genomes Project Phase 3 reference panel, filtered for European ancestry samples, with default parameters. Prior to association testing, genotype data were converted from VCF to IMPUTE format. Variants with an imputation quality score (DR^2^) < 0.4 were excluded, because higher values indicate well‐imputed variants. A total of >6 million genetic variants were imputed successfully. Association analysis was performed using SNPTEST v.2.5.4 (Marchini et al., 2007) under an additive genetic model. All genomic positions were presented based on the GRCh38 (hg38) reference assembly.

### 2.5 DNA methylation analysis

DNA methylation profiling was performed on 64 subjects (43 females, aged 85.5 ± 6.1 years; 19 males, aged 82.9 ± 8.8 years) diagnosed with sarcopenia and frailty using Illumina Infinium EPIC v.2 arrays. Quality control and preprocessing of raw methylation data were conducted in R (v.4.4.2) using the SeSAMe package (v.1.24.0) (Zhou et al., 2018). A stringent quality control threshold was applied: probes with detection *p*‐values ≤ 0.01 were retained for analysis, whereas those failing this criterion in >20% of samples were removed. Probes containing SNPs, cross‐hybridizing probes and probes located on sex chromosomes were excluded. Samples with >10% of low‐confidence probes were also removed.

To assess associations between methylation levels and LMI, β values were transformed into *M* values, which are more appropriate for differential methylation analysis (Haghshenas et al., 2020). Males and females were analysed separately to avoid sex‐related confounding, given known sex differences in methylation patterns (Singmann et al., 2015). Associations between methylation levels and the continuous LMI variable were tested using the DMRcate package (v.3.2.1) (Peters et al., 2024), applying a false discovery rate (FDR) threshold of <0.05. To validate the results from DMRcate, an additional set of algorithms implemented in SeSAMe was used to identify probes, segments and genes significantly associated with LMI. Functional enrichment analysis of significant probes and data visualization were also conducted using SeSAMe. All models included age as a covariate. Associations were evaluated at both the single‐probe level and at the level of differentially methylated regions (DMRs), where adjacent CpG sites with coordinated changes were merged.

Finally, enrichment analysis of functional genomic elements associated with significant probes was performed. Genes identified as associated with LMI through methylation signals were analysed further using the gprofiler2 package (v.0.2.3) (Kolberg et al., 2020) to determine enriched biological annotations.

### 2.6 Measurement of relative telomere length

Relative telomere length was measured using quantitative real‐time PCR and quantified as the telomere‐to‐single‐copy gene (T/S) ratio, as described by Cawthon (2002). The T/S ratio reflects the relative copy number of telomeric repeats (T) to that of a single‐copy gene (S), normalized to a reference DNA sample. The human β‐globin gene was used as the single‐copy reference, and a pooled mix of non‐study DNA samples served as the reference DNA. The T/S ratio was calculated using the following formula:

$$T/S\;\mathrm{ratio}=E{\left(tel\right)}^{Ct}$$

The T/S ratio represents relative telomere length, calculated as the ratio of telomere repeat copy number (T) to a single‐copy reference gene (S). *Ct* denotes the PCR cycle threshold, and E the amplification efficiency of the respective primer set.

Quantitative real‐time PCR was performed according to a previously published protocol (Joglekar et al., 2020), with modifications, using the 7900HT Fast Real‐Time PCR System (Applied Biosystems, USA). Modifications included an increased reaction volume of 20 µL and adjusted primer concentrations: 0.8 µM for Telomere A and 1.5 µM for Telomere B. Telomere and human β‐globin gene amplifications for each sample were performed on the same plate in the following thermal cycling conditions: 95°C for 10 min, followed by 35 cycles of 95°C for 15 s and 56°C for 2 min. A melt curve analysis was included at the end of each run to verify primer specificity. All reactions were carried out in duplicate within the same plate.

### 2.7 Statistical analysis

Data normality was assessed using the Shapiro–Wilk test, and the results indicated a non‐normal distribution. Continuous variables were reported as the median and interquartile range (25th–75th percentile), whereas categorical variables were presented as frequencies (number and percentage). Group differences between males and females in univariate analyses were assessed using the Kruskal–Wallis test for continuous data and the χ^2^ test for categorical data. Associations between LMI and various parameters were evaluated using Spearman's rank correlation. Linear regression analysis was performed to assess the impact of physical parameters on LMI. Statistical analyses were conducted using IBM SPSS Statistics for Windows, v.20 (IBM Corp., Armonk, NY, USA). Owing to the non‐normal distribution of T/S values, data were log‐transformed to achieve homoscedasticity and approximate a normal distribution of residuals. Differences in mean T/S values were analysed using analysis of covariance (ANCOVA), adjusting for age, sex, smoking status and PASE score. Logistic regression models were used to estimate the likelihood of sarcopenia or frailty, with age and sex included as covariates. Linear regression analyses were used to examine associations between the T/S ratio and phenotypic parameters of sarcopenia and frailty, with adjustments for age, sex and their interactions with the T/S ratio. These analyses were performed using R Studio (v.4.3.1). Statistical significance was defined as *p* < 0.05.

### 2.8 Genes overlapping genomic regions of proximal methylated CpGs and GWAS SNPs

The SeSAME DML function fits each DNA methylation site to a linear model, performing slope and goodness‐of‐fit tests. We applied this function to test the association between lean mass index (LMI) and DNA methylation, specifying the model as ∼ LMI + age, where LMI is the main predictor and age is included as a covariate. Analyses were conducted separately for males and females. Neighbouring CpGs showing consistent methylation variation with respect to LMI were merged using the SeSAME DMR function, which computes multiple‐testing‐adjusted *p*‐values for each segment. Only segments with adjusted *p* < 0.05 were retained. Next, we identified GWAS SNPs closest to or overlapping these methylated regions using the bed_closest function from the R package valr (Riemondy et al., 2017). SNPs within ±20 kbp of the significant methylated regions (Villicana et al., 2023) were considered, based on the estimated regulatory distance between SNPs and their target CpG islands. Finally, we annotated genes overlapping these proximal methylated CpGs and GWAS SNPs (both significantly associated with LMI, *p* < 0.05) using the Illumina EPIC v.2 manifest.

## 3 RESULTS

### 3.1 Descriptive characteristics and inter‐group comparisons

Key descriptive characteristics of the study population are presented in Table 1.

**TABLE 1:** Basic descriptive characteristics of the study population (median and interquartile range: 25th–75th percentile).

Characteristics	All participants (*n* = 204)	Control subjects (*n* = 82)	Subjects with sarcopenia and frailty (*n* = 62)	*p*‐value
Age, years	83 (77−87)	82 (79−84.25)	86 (82−89.25)	<0.001
Female, *n* (%)	161 (78.9)	65 (79.3)	40 (64.5)	0.001
Diseases, *n*	7 (4−9)	4 (3−6.25)	8 (6−10)	<0.001
Level of education, *n* (%)				0.472
Elementary	1 (0.5)	1 (1.2)	0	
Lower secondary	4 (2)	2 (2.4)	1 (1.6)	
Upper secondary	41 (20.1)	10 (12.2)	17 (27.4)	
Special secondary	27 (13.2)	10 (12.2)	6 (9.7)	
Post‐secondary	46 (22.5)	18 (22)	16 (25.8)	
Higher non‐university	23 (11.3)	8 (9.8)	6 (9.7)	
Higher university	62 (30.4)	33 (40.9)	16 (25.8)	
Smoking status, *n* (%)				0.092
Never smoked	163 (79.9)	62 (75.6)	47 (75.8)	
Used to smoke	35 (17.2)	16 (19.5)	14 (22.6)	
Currently smoking	6 (2.9)	4 (4.9)	1 (1.6)	
Alcohol consumption, *n* (%)				<0.001
Does not drink	159 (77.9)	50 (61)	56 (90.3)	
Drinks	45 (22.1)	32 (39)	6 (9.7)	
Medications, *n*	6 (4−8)	5 (3−7)	6 (3.75−8)	0.536
Polypharmacy, *n* (%)	113 (55.4)	37 (45.1)	32 (51.6)	0.008
MNA, score	11 (9−14)	14 (12−14)	8 (7−10)	<0.001
GDS, score	3 (0−5)	1.5 (0−3)	4 (0−6)	<0.001
MMSE, score	27 (24−29)	28 (27−30)	24 (23−26)	<0.001
Height, cm	167 (163−172)	167.75 (164−172.25)	169 (163.5−176.72)	0.005
Weight, kg	67.3 (59.7−78.5)	73.3 (64.07−82)	61.4 (53.8−77.1)	<0.001
BMI, kg/m^2^	24 (21.59−26.97)	25.24 (23.96−27.6)	21.55 (19.73−24.71)	<0.001
Dynamometry, kg	14 (12−23.5)	21 (14‐29.25)	12 (9.5−18.5)	<0.001
Gait speed, m/s	0.68 (0.53−0.82)	0.83 (0.71−0.95)	0.52 (0.47−0.59)	<0.001
SPPB, points	8.5 (6−10)	10 (9−12)	6 (4−7)	<0.001
PASE, score	35 (27−76)	76 (51−105)	27 (25−30)	<0.001
ADL, score	6 (4−6)	6 (4−6)	3 (1−4.25)	<0.001
IAD, score	5 (2−8)	8 (7−8)	2 (1−2.25)	<0.001
Lean mass, kg	39.01 (32.96−43.31)	41.95 (39.57–46.22)	34.72 (30.2–39.64)	<0.001
Appendicular lean mass, kg	18.09 (15.31−21.63)	20.97 (18.68–22.93)	15.51 (14.5–19.53)	<0.001
Fat mass, kg	24.6 (20.18−31.97)	26.97 (22.17–34.24)	21.64 (18.45–28.44)	0.02
Lean mass index, kg/m^2^	13.62 (12.08–15.55)	15.2 (13.62–16.77)	12.38 (11.23–13.29)	<0.001
Simple reaction time, ms	326 (285–405)	285 (258–324)	406 (358–478)	<0.001
Complex reaction time, ms	617 (527–782)	509 (448–578)	781 (673–913)	<0.001
10 s tapping test, count	51 (45.75–56)	55 (51–60)	44 (41–49)	<0.001
60 s tapping test, count	253 (217–298)	299 (259–323)	215.5 (188–239.75)	<0.001
Modified Borg Scale, score	7 (5–8)	7 (4–7)	7 (4.25–8)	0.036
Muscle oxygenation during exercise, $S_{mO_{2}}$ (%)	60.48 (35.88–85.08)	68.06 (48.31–87.81)	26.00 (14.86–37.14)	<0.001

*Note*: The *p*‐value was calculated using the Kruskal−Wallis test when comparing control, sarcopenic, frail, and sarcopenic and frail participants.

Abbreviations: ADL, activities of daily living; BMI, body mass index; GDS, Geriatric Depression Scale; IADL, instrumental activities of daily living; MMSE, Mini Mental State Examination; MNA, Mini Nutritional Assessment; PASE, Physical Activity Scale for the elderly; $S_{mO_{2}}$, muscle oxygen saturation; SPPB, Short Physical Performance Battery.

When comparing groups, individuals with sarcopenia and/or frailty were older, had a higher number of comorbidities and reported more frequent alcohol consumption in comparison to the control group (Table 1). No significant differences were observed between groups regarding education level or smoking status. The total number of medications taken did not differ significantly between groups; however, polypharmacy was more prevalent among participants with sarcopenia and/or frailty.

Cognitive function was within the normal range in all groups, although lower Mini‐Mental State Examination scores were observed in the sarcopenia and/or frailty groups. Depressive symptoms were also within the normal range across all groups, but higher GDS scores were found among those with sarcopenia and/or frailty. The risk of malnutrition was noted in the sarcopenia and/or frailty groups, whereas participants in the control group exhibited normal nutritional status.

Participants with sarcopenia and/or frailty demonstrated significantly reduced muscle strength, slower gait speed, lower physical performance and decreased levels of physical activity in comparison to the control group. Additionally, impairments in everyday activities were more pronounced in the sarcopenia and/or frailty group. Psychomotor speed parameters also differed between groups; participants in the sarcopenia and/or frailty groups were slower in both simple and complex reaction time tasks, in addition to the tapping test.

The study revealed that $S_{mO_{2}}$ during the resting phase differed significantly between the control and sarcopenia/frailty groups (77.88% ± 16.60% vs. 35.09% ± 7.43%, respectively), as did $S_{mO_{2}}$ at the end of standardized physical exertion (68.06% ± 19.75% vs. 26.00% ± 11.14%) and after 5 min of recovery (77.55% ± 16.18% vs. 46.09% ± 11.21%) (*p*  <  0.001). However, the relative decrease in $S_{mO_{2}}$ during exercise was similar in both groups, ∼9%–10%. Interestingly, during recovery, the sarcopenia and/or frailty group reached an $S_{mO_{2}}$ level that was 11% higher than their pre‐exercise value.

Furthermore, body composition differed significantly between groups. Participants in the control group had greater muscle and fat mass, in addition to a higher LMI, compared with those in the sarcopenia and/or frailty groups. Across all participants, LMI was associated with age, number of diseases and medications, physical activity, physical performance, nutritional status and handgrip strength (Table 2). No significant association was found between LMI and depressive symptoms (*p* = 0.07).

**TABLE 2:** Lean mass index in relationship to other phenotypic parameters in all participants.

Parameters	Lean muscle index^a^	*p*‐value
Age	−0.202	0.004
Number of diseases	−0.313	<0.0001
Number of medications	−0.143	0.041
PASE score	0.342	<0.001
SPPB score	0.184	0.009
MNA score	0.346	<0.001
Dynamometry	0.495	<0.001

*Note*: Abbreviations: MNA, Mini Nutritional Assessment; PASE, Physical Activity Scale for the Elderly; SPPB, Short Physical Performance Battery.

^a^ Spearman's correlation coefficient (*r*).

### 3.2 Phenotypic associations

The likelihood of developing sarcopenia and/or frailty increases by 12.5% with each additional year of age {odds ratio (OR) = 1.125, 95% confidence interval (CI) [1.033, 1.228], *p* = 0.007}. Specifically, the risk of developing sarcopenia rises by 13.8% for each additional year of age (OR = 1.138, 95% CI [1.042, 1.251], *p* = 0.005), while the risk of frailty increases by 15.5% for every additional year of age (OR = 1.155, 95% CI [1.046, 1.283], *p* = 0.005). Participants with both sarcopenia and frailty experienced the highest annual increase in the risk of developing these conditions (OR = 1.189, 95% CI [1.062, 1.347], *p* = 0.004).

In the male group, LMI was positively associated with physical activity (*r* = 0.306, *p* = 0.048), physical performance (*r* = 0.399, *p* = 0.008), nutritional status (*r* = 0.440, *p* = 0.003) and handgrip strength (*r* = 0.414, *p* = 0.006). No significant associations were observed between LMI and age (*p* = 0.662), number of diseases (*p* = 0.057), number of medications (*p* = 0.635) or depressive symptoms in the male group.

In the female group, LMI showed a negative association with age (*r* = −0.216, *p* = 0.006) and number of diseases (*r* = −0.329, *p* < 0.001), and a positive association with nutritional status (*r* = 0.329, *p* < 0.001), physical activity (*r* = 0.353, *p* < 0.001) and handgrip strength (*r* = 0.510, *p* < 0.001). No significant associations were found between LMI and the number of medications (*p* = 0.059), depressive symptoms (*p* = 0.321) or physical performance (*p* = 0.101) in this group. Additionally, a higher level of education was positively associated with LMI (*r* = 0.176, *p* = 0.025).

Linear regression analysis revealed that LMI was positively associated with handgrip strength and frequency of movement and negatively associated with exercise tolerance (Table 3). Furthermore, the linear regression model indicated significant associations between cognitive function, depressive symptoms and LMI. A higher Mini‐Mental State Examination score was associated with greater LMI (β = 0.166, 95% CI [0.028, 0.304], *p* = 0.019), whereas a higher GDS score was associated with lower LMI (β = −0.157, 95% CI [−0.293, −0.021], *p* = 0.023).

**TABLE 3:** Linear regression model between lean mass index and handgrip strength, frequency of movement and exercise tolerance.

Model	*β*	95% CI	*p*‐value
Dynamometry	0.124	0.087–0.161	<0.001
60 s tapping test	0.025	0.014–0.037	<0.001
Modified Borg Scale	−0.407	−0.594–0.291	<0.001

*Note*: Model adjusted for age, sex, physical activity, number of diseases, nutritional status, depressive symptoms, education level, smoking status and alcohol consumption. β, effect size

### 3.3 GWAS

Before genotype imputation, an initial GWAS was conducted comparing cases and controls, where cases were defined as individuals with sarcopenia, frailty or both conditions. No genome‐wide significant associations (defined as *p* < 5 × 10^−8^) were identified in any of the case–control comparisons.

Subsequent GWAS analyses using imputed genotype data were conducted to examine associations with specific phenotypic traits related to sarcopenia and frailty, including handgrip strength, gait speed, exhaustion, physical activity, weight loss and LMI, as defined earlier. Two SNPs, rs75652203 (located near *CLIC5*) and rs17102732 (near *GHITM*), demonstrated significant associations with handgrip strength. The raw *p*‐values for these associations were 8.13 × 10^−12^ (Bonferroni‐corrected *p* = 1.42 × 10^−6^; permutation‐based *p* = 0.0004) and 3.37 × 10^−9^ (Bonferroni‐corrected *p =* 0.000587; permutation‐based *p* = 0.0194), respectively.

Additionally, four SNPs [rs3744589 (in *ACACA*), rs850577 (in *KLHL32*), rs2850114 (in *CLDN14*) and rs8066532 (near *SLC39A11*)] showed suggestive associations with handgrip strength, with raw *p*‐values below the suggestive threshold of 1 × 10^−5^. However, these associations did not remain significant after permutation testing and should therefore be interpreted with caution.

Likewise, the SNPs rs60001950 (near *TBL1XR1*) and rs10903128 (near *RUNX3*) were suggestively associated with walking pace; rs12084520 (in *KIF26B*) and rs10733084 (near *KCNT2*) with LMI; and rs76706220 (near *CNTN5*) and rs71506686 (near *AGTPBP1*) with weight loss. However, none of these associations reached statistical significance after permutation testing.

Finally, 12 SNPs previously linked to sarcopenia (Yang et al., 2025) were replicated (*p* < 0.05) in relationship to LMI: *BOK* rs76993203, *VAMP5* rs1374370, *TMEM18* rs12714414, *SFMBT1* rs36033494, *BANK1* rs13136118, *TET2* rs2647239, *FOXO3* rs9384679, *L3MBTL3* rs13209574, *ZFAT* rs13267329, *CEP57* rs35793328, *PCGF2* rs1985352 and *MC4R* rs66922415 (Table 4). Of these SNPs, only one (*TMEM18* rs12714414) remained statistically significant after Bonferroni correction for multiple testing (*p *≤ 0.00016; 0.05/307 sarcopenia‐related SNPs).

**TABLE 4:** Replication of sarcopenia‐associated single nucleotide polymorphisms in relationship to lean mass index.

Gene or nearest gene	SNP	Alleles	Protective allele	β_1_ (LMI)	*p*_1_‐value (LMI)	β_2_ (sarcopenia)	*p*_2_‐value (sarcopenia)
*BOK*	rs76993203	G/A	G	0.935	0.0466	−0.0104	1.15 × 10^−9^
*VAMP5*	rs1374370	G/A	A	2.036	0.0014	−0.0112	1.97 × 10^−9^
*TMEM18*	rs12714414	T/C	T	2.783	0.000031^a^	−0.0187	1.46 × 10^−17^
*SFMBT1*	rs36033494	C/T	C	1.182	0.0407	−0.0129	1.50 × 10^−13^
*BANK1*	rs13136118	G/A	G	1.296	0.0352	−0.0127	2.25 × 10^−9^
*TET2*	rs2647239	A/G	A	0.912	0.0385	−0.0146	1.18 × 10^−18^
*FOXO3*	rs9384679	C/T	C	1.540	0.0104	−0.0110	1.90 × 10^−10^
*L3MBTL3*	rs13209574	G/T	G	1.431	0.0450	−0.0204	1.40 × 10^−12^
*ZFAT*	rs13267329	G/A	A	1.646	0.0082	−0.0200	4.27 × 10^−17^
*CEP57*	rs35793328	G/T	T	1.259	0.0134	−0.0100	4.48 × 10^−8^
*PCGF2*	rs1985352	G/A	A	1.615	0.0248	−0.0164	1.29 × 10^−17^
*MC4R*	rs66922415	A/G	G	1.299	0.0291	−0.0286	5.99 × 10^−58^

*Note*: Abbreviations: β_1_, effect size of the protective allele in the present study; β_2_, effect size (in relationship to protective allele) reported by Yang et al. (2025); LMI, lean mass index; *p*_2_, *p*‐value for sarcopenia risk by Yang et al. (2025); protective allele, the allele associated with a lower risk of sarcopenia in the study by Yang et al. (2025) and with higher LMI in the present study; SNP, single nucleotide polymorphism.

^a^ *p*_1_ < 0.00016; statistically significant association with LMI after Bonferroni correction.

### 3.4 Methylome‐wide association study

Quality control filtering resulted in 60 samples (42 females and 18 males with sarcopenia and/or frailty) and 822 579 probes retained for analysis. The DMRcate association testing between methylation level and LMI, using age as a covariate at a 0.05 FDR, did not return individually significant probes in the male group. In the female group, seven individually significant probes were identified, covering the *CCT5* gene locus extending by 420 bp (chr5:10250050–10250469) (*p* = 4.59 × 10^−26^) (Cerezo et al., 2025; Peters et al., 2024).

Further analysis using SeSAMe identified 154 individual probes significantly associated with LMI in males, spanning 26 genomic regions, after Benjamini–Hochberg adjustment (BH *p*‐value < 0.05). In the female group, 85 probes were significantly associated with LMI, spanning 21 genomic regions (BH *p*‐value < 0.05). Details are presented in the Supplementary Data spreadsheet. We also analysed how probes significantly associated with LMI in males and females are enriched in different databases of functional genomic elements (Figure S1). The enrichment results show that the significant probes are most abundantly distributed across transcription factor binding sites (TFBS), histone modification and protein coding metagenes (PCmetagenes) databases. PCmetagenes refers to genes within the metagenome that are predicted to encode proteins, which are used to study functions of short sequences from metagenome sequencing data. The enrichment results indicate that the identities of elements enriched by probes significantly associated with LMI differ between males and females.

Methylation of transcription factor (TF) binding sites can either enhance or inhibit TF binding, thus impacting gene expression. Significantly enriched TFBS in males and females are illustrated in Figure S2. The most significantly enriched TF in females was LEO1, a subunit of the RNA polymerase‐associated factor 1 complex. LEO1 plays a crucial role in transcription elongation, histone turnover and DNA repair, particularly in response to transcription‐blocking DNA damage. It is essential for the dynamic regulation of heterochromatin and gene expression during cellular quiescence. Although LEO1 is broadly involved in transcriptional elongation, its enrichment in our analysis might reflect transcriptional changes driven by inflammaging, a chronic low‐grade inflammatory state associated with ageing and immune dysfunction. In males, the most significantly enriched TF was Epstein–Barr virus (EBV) nuclear antigen 2 (EBNA2), encoded by the Epstein–Barr virus. It plays an important role in B‐cell immortalization and viral latency. Regulation of cellular gene expression by EBNA2 might affect the risk of certain cancers, including lymphomas, and autoimmune conditions, such as multiple sclerosis.

We tested which genes were most significantly enriched by the probes whose methylation levels are associated with LMI. The region of interest comprises the gene body, extended 100 bp upstream of the gene transcription start site (TSS). In males, 31 such genes were identified (Figure S3). In females, 26 such genes were identified (Figure S4). The results indicate different genes associated with LMI through methylation in males and females. Table S1 presents the genes enriched (FDR < 0.01) by probes whose methylation levels are significantly associated with LMI in both males and females. Table S1 also summarizes the gene methylation status (hyper‐ or hypo‐) and the phenotypic associations of the gene recorded in the Genome‐Wide Association Studies Registry (Cerezo et al., 2025). Most of these genes are associated with rheumatoid arthritis, anthropometric body characteristics, lipid metabolism, inflammatory processes and the immune system. The genes *GNAS* and *SCGN* are associated with appendicular lean mass and body lean mass. Overall, genes enriched by methylated probes associated with LMI in sarcopenia patients collectively play important roles in the immune system; some belong to the major histocompatibility complex (MHC). Other genes are involved in wide‐scale regulatory processes and chromatin dynamics (Tables S2 and S3 summarize gene functions).

We performed an enrichment analysis of annotations using gprofiler2 (Kolberg et al., 2020) for the male and female gene lists enriched by the methylated probes significantly associated with LMI. The male gene list showed significant enrichment results (BH *p*‐value < 0.05), as detailed in Table S1. The male gene list was enriched by one annotation from the CORUM database: the AIRE homodimer complex. The female gene list was enriched with multiple annotations. The Gene Ontology annotations in the biological process and molecular function classes collectively point to antigen processing and the MHC protein complex, in addition to antigen binding. Enriched Reactome pathways included MHC class II antigen presentation, interferon gamma signalling and translocation of ZAP‐70 to the immunological synapse, highlighting the involvement of female genes associated with LMI through methylation in inflammatory and immune processes. Likewise, pathways enriched in KEGG collectively represent immune processes and disorders associated with the immune system (Table S1).

### 3.5 Association between telomere length and phenotypes

Individuals with sarcopenia and/or frailty exhibited significantly shorter telomeres (mean: 0.286, 95% CI [0.261–0.312]) compared with community‐dwelling older adults in the control group (mean: 0.365, 95% CI [0.320–0.409]; *p* = 0.003) (Table 5). After adjusting for age and sex, significant differences in relative telomere length (T/S ratio) remained only in the sarcopenia and frailty group (mean: 0.267, 95% CI [0.225–0.308]) compared with the control group (mean: 0.350, 95% CI [0.309–0.392]; *p* = 0.006).

**TABLE 5:** Relative telomere length of participants.

	Relative telomere length (T/S ratio)			
Groups	Unadjusted mean (95% CI)	*p*‐value	Adjusted mean (95% CI)^a^	*p*‐value
Control subjects (*n* = 75)	0.365 (0.328–0.401)		0.350 (0.309–0.392)	
Sarcopenia and/or frailty (*n* = 122)	0.286 (0.261–0.312)	0.003	0.296 (0.263–0.329)	0.066
Sarcopenia (*n* = 31)	0.287 (0.256–0.318)	0.005	0.300 (0.262–0.338)	0.136
Frailty (*n* = 29)	0.285 (0.225–0.344)	0.025	0.296 (0.232–0.360)	0.137
Sarcopenia and frailty (*n* = 62)	0.250 (0.210–0.290)	<0.001	0.267 (0.225–0.308)	0.006

*Note*: Abbreviations: CI, confidence interval; T/S, telomere/single‐copy gene ratio.

^a^ Adjusted for age and sex.

The T/S ratio was not found to be a significant independent predictor of sarcopenia or frailty. However, an interaction was observed between T/S ratio and physical activity level: longer telomeres were associated with higher PASE scores (β = 461.0, 95% CI [202.2–719.9], *p* < 0.001), although this relationship diminished with advancing age. This association was more pronounced among frail older adults (β = 582.9, 95% CI [265.4–903.4], *p* = 0.0004) in comparison to those with sarcopenia (β = 446.7, 95% CI [168.4–724.9], *p* = 0.002). Moreover, frail individuals with longer telomeres tended to use fewer medications (β = −2.89, 95% CI [−5.18 to −0.61], *p* = 0.01).

Longer telomeres (bigger T/R ratio) were also associated with higher total haemoglobin concentration in muscle tissue after 5 min of recovery (β = 6.94, 95% CI [2.26–11.63], *p* = 0.005). Additionally, in frail participants, the T/R ratio was associated with total haemoglobin concentration at the end of the standardized physical load (β = 9.2, 95% CI [3.0–15.4], *p* = 0.005). No significant associations were observed between the T/R ratio and exhaustion, gait speed, handgrip strength, weight loss or LMI.

Furthermore, the T/R ratio was associated with 49 differentially methylated CpG sites at an FDR of <0.05. These CpG sites mapped to seven genetic loci (*FPGT*, *LRRIQ3*, *TNFSF9*, *ZDHHC14*, *LINC00240*, *MPL* and *SH3RF3*) and overlapped with regulatory regions identified by ENCODE and H3K27Ac histone mark elements. These genes are collectively linked to key phenotypic traits such as smoking, alcohol consumption, educational attainment, mental health, depressive symptoms, body mass index, body fat mass and bone mineral density.

### 3.6 Genes overlapping genomic regions of proximal methylated CpGs and GWAS SNPs

Four genes in females and 16 in males were found to be associated with methylated regions proximal (within 20 kb) to significant GWAS SNPs, both of which were linked to LMI. The results are presented in Table 6. Among these, nine genes in males (*EXOC3L2*, *KIFC3*, *PEX5L*, *AIRE*, *KCNA4*, *PRAM1*, *SMG6*, *SCGN* and *PPP1R18*) and two in females (*CCT5* and *ATPSCKMT*) had at least one probe whose methylation level was significantly associated with age and thus were not uniquely associated with sarcopenia and frailty phenotypes. In both males and females, two genes, *UNC45A* and *HDDC3*, were uniquely associated with LMI, where methylated probes were in close proximity to two GWAS SNPs (rs2601189 and rs2589944). Complete details on genes overlapping methylated probes and GWAS SNPs can be found in Table S4.

**TABLE 6:** Genes overlapping genomic regions of proximal methylated CpGs and SNPs, both associated with lean mass index at a 95% significance level (*p *< 0.05).

Gene with methylated CpGs (locations of methylated probes in functional gene elements)	SNP (chromosome: position)	Reference allele	Alternative allele	Genomic coordinates of the segment with methylated probes	List of methylated probes	Distance between the methylated segment and SNP^a^ (bp)
Females						
*UNC45A* (TSS200), *HDDC3* (exon_4)	rs2601189 (chr15: 90929874)	A	G	chr15: 90929829−90930340	cg20800831_TC11	0
*UNC45A* (TSS200), *HDDC3* (exon 4)	rs2589944 (chr15: 90938720)	C	T	chr15: 90929829−9093034	cg20800831_TC11	8381
*CCT5* (TS200)^b^, *ATPSCKMT* (TSS1500)	rs116055676 (chr5: 10227350)	T	C	chr5: 10250166−10251141	cg11101846_TC21	−22 816
Males						
*UNC45A* (TSS200), *HDDC3* (exon 4)	rs2601189 (chr15: 90929874)	A	G	chr15: 90929829−90930340	cg20800831_TC11	0
*UNC45A* (TSS200), *HDDC3* (exon 4)	rs2589944(chr15:90938720)	C	T	chr15: 90929829−90930340	cg20800831_TC11	8381
*PPP1R18* (exon 1, exon 2)^b^	rs4713355 (chr6: 30685810)	C	T	chr6: 30685630−30685773	cg03052182_BC21, cg12105190_BC21, cg12247101_TC11, cg25980484_BC21	38
*SCGN* (TSS200)^b^	rs72840525 (chr6: 25651703)	T	G	chr6: 25652153−25652304	cg04200224_TC11, cg10721149_TC11, cg13586599_BC21, cg13834623_BC11, cg15562220_BC11	−450
*SMG6* (TSS200, TS1500)^b^	rs62067014 (chr17: 2265592)	G	A	chr17: 2266208−2266696	cg02976056_BC21, cg07350732_TC21, cg09409898_TC21, cg22037995_BC21, cg23218897_TC21	−616
*TRIM39* (exon 3)	rs3094621 (chr6: 30328753)	C	T	chr6: 30329397−30329851	cg19047804_TC21	−644
*PRAM1* (exon 2)^b^	rs34350946 (chr19: 8495115)	T	C	chr19: 8496141−8499790	cg01995393_TC21, cg08474813_TC21, cg14242207_TC21, cg14324305_TC21, cg23055276_TC21	−1026
*ZNF155* (TSS1500, 5UTR, exon 1, TSS200)	rs11665812 (chr19: 43986023)	T	C	chr19: 43983969−43984732	cg10590767_TC11, cg10754510_BC21, cg20451226_TC11, cg23456212_BC11, cg24582868_TC21, cg24582869_BC21, cg27309253_BC21	1292
*KCNA4* (TSS1500, TSS200)^b^	rs142615395 (chr11: 30015403)	C	A	chr11: 30017068−30017251	cg03506489_BC21, cg13161658_BC11, cg15044957_BC11, cg15310492_TC11	−1665
*DUSP22* (TSS1500, TSS200, 5UTR, exon 1)	rs3800239 (chr6: 295085)	C	A	chr6: 291687−293286	cg01171360_TC11, cg03395511_BC21, cg05064044_BC21, cg11235426_TC21, cg15383120_TC21, cg17876578_TC21, cg18110333_BC11, cg21548813_BC21, cg26668828_BC11	1800
*CALHM2* (TSS1500)	rs115878498 (chr10: 103447267)	A	G	chr10: 103452724−103452736	cg09336982_BC11, cg21686808_TC21, cg23175074_TC11	−5457
*C1GALT1C1L* (TSS1500, TSS200)	rs6761913 (chr2: 43665245)	C	G	chr2: 43676550−43676873	cg08726338_TC21, cg13324389_TC21, cg14454477_TC21, cg18817560_BC21, cg20563666_BC21	−11 305
*AIRE* (TSS1500, TSS200, exon 1)^b^	rs117813038 (chr21: 44298932)	C	A	chr21: 44285660−44286112	cg01323542_BC21, cg08089567_BC11, cg08089567_BC12, cg11923631_TC11, cg16501323_BC21, cg16717549_TC11, cg17356252_TC21, cg18689454_BC11, cg18689454_BC12, cg27251412_TC11	12 821
*PEX5L* (exon 1, 5UTR)^b^	rs184015719 (chr3: 180051441)	C	T	chr3: 180036745−180036828	cg02119363_TC11, cg04894619_BC11, cg13473356_TC11, cg23346462_TC11	14 614
*KIFC3* (exon 2, TSS200)^b^	rs534752446 (chr16: 57814558)	C	G	chr16: 57798088−57798319	cg00947878_BC11, cg13352096_TC11, cg13750905_TC21, cg15627357_TC11, cg26927427_TC21	16 240
*EXOC3L2* (exon 3)^b^	rs79745266 (chr19: 45215805)	C	T	chr19: 45234345−45234535	cg00449767_TC11, cg01565314_BC11, cg08882547_TC11, cg09450024_BC11	−18 540

*Note*: Abbreviations: chr, chromosome; LMI, lean mass index; SNP, single nucleotide polymorphism.

^a^ A negative distance indicates that the SNP is upstream of the methylated probe region, whereas a distance of zero or positive indicates that the SNP overlaps or is downstream of the methylated probe region.

^b^ Methylation levels in at least one probe were significantly associated with age in addition to lean mass index.

## 4 DISCUSSION

The contribution of environmental, genetic and epigenetic factors to muscle mass in older adults is not yet well established. Although significant heritability estimates for muscular characteristics have been reported and several candidate genes identified, these factors explain only a small proportion of the observed variation in strength, muscle mass and function. In this study, we applied a combined omics approach (genomics, epigenomics and telomere length) to identify interpretable molecular determinants of lean mass variation.

Initially, we found that LMI is associated with several health and environmental factors. LMI represents lean mass adjusted for height and serves as a proxy for muscle quantity. Lower LMI was associated with older age, which might reflect age‐related changes in body composition (i.e., a decline in muscle mass and an increase in fat mass). These patterns have also been observed in previous studies (Ji et al., 2023; Yamada et al., 2014).

Multimorbidity was found to impact LMI negatively. A longitudinal study of older adults in England reported that multimorbidity increases the risk of sarcopenia, which is characterized by reduced muscle strength and mass (Veronese et al., 2021). Although the exact mechanisms linking multimorbidity and reduced muscle quantity remain unclear, chronic inflammation and reduced physical activity might contribute (Barbera et al., 2025; Graham et al., 2021; Shi et al., 2023). Additionally, we observed a negative relationship between LMI and the number of medications taken. Although the exact mechanism by which polypharmacy influences muscle mass and strength is uncertain, previous studies have reported similar findings (Kinoshita et al., 2025).

Our results also revealed positive associations between LMI and handgrip strength, physical performance and physical activity. Handgrip strength and LMI are closely related, reflecting muscle strength and quantity, respectively. Physical performance and activity levels also influence muscle mass (Kitamura et al., 2021; Wang et al., 2022). Moreover, better nutritional status was associated with higher LMI. Malnutrition and sarcopenia are common geriatric syndromes that share underlying risk factors. The Global Leadership Initiative on Malnutrition recommends the inclusion muscle mass assessment in the diagnostic criteria for malnutrition (Barazzoni et al., 2022). Slight differences between male and female participants were noted in the associations between LMI and variables such as multimorbidity, polypharmacy and physical activity. These differences might be attributable to unequal sample sizes.

We also found that higher psychomotor speed was associated with greater LMI. This finding is supported by a previous study reporting a link between appendicular lean mass and psychomotor speed (Tessier et al., 2022). Loss of muscle mass might result in muscle fibre loss and neuromuscular junction dysfunction, potentially explaining this relationship (Baumgartner & Kao, 2024; Sirago et al., 2023). Furthermore, lower perceived exertion was associated with higher LMI, possibly owing to improved exercise tolerance and physical performance in individuals with greater muscle mass (Craighead et al., 2024; Lu et al., 2019).

Finally, LMI was positively associated with cognitive function in older adults. One potential mechanism involves reduced myokine secretion owing to physical inactivity. Myokines, which are secreted by skeletal muscle during exercise, can cross the blood–brain barrier and influence brain function (Oudbier et al., 2022). A negative association was also observed between LMI and depressive symptoms. Both low muscle mass and depression are influenced by physical inactivity and poor physical performance. Previous studies have confirmed the link between depressive symptoms and sarcopenia or low muscle mass (Ainsworth et al., 2023; Li et al., 2022).

Based on our results, two SNPs, rs75652203 and rs17102732, showed significant associations with handgrip strength in individuals with frailty and sarcopenia. Additionally, four SNPs, rs3744589, rs850577, rs2850114 and rs8066532, were suggestively associated with handgrip strength. Likewise, rs60001950 and rs10903128 were suggestively associated with walking pace, rs76706220 and rs71506686 with weight loss, and rs12084520 and rs10733084 with LMI.

A previous GWAS on frailty identified two variants, rs6765037 and rs7134291 (Mekli et al., 2018), neither of which showed an association with frailty in our study. In the UK Biobank cohort, GWAS researchers identified 14 genetic loci associated with frailty indices among 164 610 community‐based individuals of European ancestry aged 60–70 years (Atkins et al., 2021). Many of these loci were previously linked to traits such as body mass index, smoking initiation, cardiovascular disease, depression and neuroticism, highlighting the role of behavioural and mental health factors in the development of frailty.

Moreover, genetic variants involved in immune function might contribute to increased morbidity and physical weakness in older adults with sarcopenia (Atkins et al., 2021; Jones et al., 2020). The UK Biobank has also identified hundreds of SNPs significantly associated with lean mass, handgrip strength and walking pace. However, only 78 SNPs have been associated with phenotypes linked to sarcopenia, including tiredness, neuroticism, frequency of alcohol intake, history of falls, smoking, time spent watching television, processed meat intake, bone mineral density, cognitive performance, educational attainment and physical activity (Semenova et al., 2023). In a larger study, Yang et al. (2025) reported associations between 307 SNPs and sarcopenia. Of these, 12 SNPs were replicated in our study in relationship to LMI, showing the same direction of association.

Furthermore, we identified methylated regions significantly associated with LMI in Lithuanian patients with sarcopenia and/or frailty. Several genes were significantly enriched with methylated probes associated with LMI. Collectively, these genes are involved in inflammation and immune system processes. Age‐related immune decline (immunosenescence) and chronic low‐grade inflammation (inflammaging) are both implicated in the pathogenesis of sarcopenia and frailty. These geriatric syndromes can lead to reduced muscle mass, potentially contributing to immunosenescence. Skeletal muscle might act as a key integrator linking sarcopenia and immune senescence.

In our analysis, a hypomethylated region in *CCT5*, comprising seven probes, was significantly associated with LMI in female patients with sarcopenia. Hypomethylation might lead to overexpression of *CCT5*. The protein encoded by this gene is a molecular chaperone and a component of the chaperonin‐containing TCP1 complex (CCT), also known as the TCP1 ring complex (TRiC), which assists in the folding of various proteins, including actin and tubulin. Mutations in *CCT5* have been linked to hereditary sensory and autonomic neuropathy with spastic paraplegia. Overexpression of this gene has also been implicated in tumorigenesis (Wu & Peng, 2024).

Moreover, the most significantly enriched methylation‐associated TF in females was LEO1, and in males, EBNA2. The presence of EBNA2 enrichment might suggest viral reactivation or persistent immune activation, which could intersect with age‐related immune remodelling. Although LEO1 is broadly involved in transcriptional elongation, its enrichment in our analysis (particularly alongside immune‐related genes) might reflect biologically meaningful processes in ageing, notably inflammaging. The transcriptional signature observed in our study might therefore capture underlying inflammatory mechanisms. Nevertheless, we acknowledge that the predominance of immune cells in peripheral blood samples might influence gene enrichment patterns. Future studies using tissue‐specific samples (e.g., skeletal muscle) will be essential to refine these associations and improve mechanistic insight.

The identification of genes overlapping methylated CpG regions proximal to GWAS SNPs associated with LMI provided valuable insights into potential epigenetic mechanisms underlying sarcopenia and frailty. Notably, *UNC45A* and *HDDC3* emerged as key candidates in both sexes, with methylation sites near SNPs (rs2601189 and rs2589944) uniquely linked to lean mass, independent of age‐related methylation changes. This suggested a potential role for these genes in muscle maintenance and sarcopenia pathogenesis. *UNC45A* encodes a regulatory component of the progesterone receptor/heat shock protein 90 (HSP90) chaperoning complex, which is involved in the assembly and folding of the progesterone receptor. This process is essential for normal cell proliferation and myosin accumulation during muscle cell development. Mutations in *UNC45A* are associated with osteoatohepatoenteric syndrome (MedGen UID: 1785846), characterized by bone fragility, hearing loss, cholestasis and congenital diarrhoea. *HDDC3* (HD domain‐containing 3) is predicted to exhibit guanosine‐3′,5′‐bis(diphosphate) 3′‐diphosphatase activity. However, neither *HDDC3* nor the SNPs rs2601189 and rs2589944 have reported associations with sarcopenia, frailty or other phenotypes in major genomic databases. Other genes in males exclusively associated with LMI in our study included *TRIM39*, *ZNF155*, *DUSP22*, *CALHM2* and *C1GALT1C1L*. No phenotypic associations for these genes were found in genomic databases. In females, *CCT5* and *ATPSCKMT* exhibited methylation levels significantly associated with both age and LMI. *CCT5* is linked to hereditary sensory neuropathy with spastic paraplegia, whereas *ATPSCKMT* is associated with chronic widespread pain. In males, *EXOC3L2*, *KIFC3*, *PEX5L*, *AIRE*, *KCNA4*, *PRAM1*, *SMG6*, *SCGN* and *PPP1R18* showed methylation levels associated with both age and LMI. Variants in *EXOC3L2* may be linked to Alzheimer's disease, and mutations in *AIRE* cause autoimmune polyendocrinopathy with candidiasis and ectodermal dystrophy, a rare autosomal recessive disorder. *KCNA4* is associated with microcephaly, cataracts, intellectual disability and dystonia with striatal abnormalities. Sequence variants in *PRAM1* influence adult human height diversity, and variants in *SMG6* are linked to schizophrenia and coronary heart disease. Overall, integration analysis of methylation and GWAS data indicated that *UNC45A* might be associated with sarcopenia in the Lithuanian elderly study cohort.

In addition, we found a significant association between telomere length and methylation levels in genes linked to lifestyle traits that might influence the risk of sarcopenia and frailty in the Lithuanian elderly population. Specifically, telomere length was associated with 49 differentially methylated CpG sites.

The present study demonstrated significantly longer telomere lengths in the control group. Patients with frailty syndrome exhibited shorter telomeres in comparison to both patients with sarcopenia and community‐dwelling older adults in the control group. The likelihood of developing sarcopenia and/or frailty increased by 12.5% with each additional year of age. Previous studies have shown that telomere length is influenced not only by age but also by lifestyle factors, psychological status and the presence of chronic diseases (Cederholm, 2015; Kameda et al., 2020; Kmiołek et al., 2023).

In the present study, longer telomere length was associated with higher levels of physical activity and greater total haemoglobin concentration in muscle. However, no significant associations were observed between telomere length and exhaustion, gait speed, handgrip strength, weight loss or LMI. According to other authors, molecular differences in muscle loss might stem from distinct catabolic processes occurring in frailty (general muscle atrophy and weakness) versus sarcopenia (loss of specific muscle mass and function) (Cederholm, 2015; Kmiołek et al., 2023).

It is important to note that in our cohort, telomere length was assessed using T/S ratios, which reflect the relative abundance of telomeric repeats rather than absolute telomere length. This metric, derived from qPCR‐based analysis, might be influenced by genomic alterations such as telomere fusion, variant repeat sequences or chromosomal rearrangements. However, a decreased T/S ratio typically indicates a reduction in telomeric repeat copy number and is widely interpreted as a marker of telomere shortening. This decline might arise from physiological processes, such as replicative senescence, whereby telomeres progressively shorten with each cell division, or from environmental stressors, such as oxidative damage, which accelerate telomere attrition. Furthermore, pathophysiological conditions (including impaired telomerase activity or chronic DNA damage) can compromise telomere maintenance, contributing to lower T/S ratios.

Additionally, we would like to emphasize that our study had certain limitations. The most notable was the relatively small sample size. Additionally, we were unable to identify biomarkers that distinctly differentiate sarcopenia from frailty syndrome. Although peripheral blood is a practical and widely used source for DNA methylation studies, we acknowledge that it might not fully reflect tissue‐specific epigenetic signatures relevant to sarcopenia and frailty, which primarily affect skeletal muscle and neuromuscular systems. Nevertheless, previous research has shown that whole blood methylation profiles can yield biologically meaningful associations, particularly those related to systemic inflammation, immune function and ageing‐related pathways. Future studies incorporating tissue‐specific methylation data (e.g., muscle biopsies) might help to refine these findings and improve mechanistic insights. Moreover, future genome‐ and methylome‐wide association studies involving larger cohorts of individuals with sarcopenia and frailty could provide valuable insights. Such studies might help to identify associations with skeletal muscle characteristics (e.g., LMI) and reveal group‐specific molecular differences, potentially leading to more precise diagnostic tools and targeted interventions for frailty and/or sarcopenia.

## 5 CONCLUSION

Frailty and sarcopenia are linked to higher morbidity and mortality, although their broad definitions might hinder pinpointing specific muscle phenotypes and genetic profiles. In 204 elderly participants, LMI was correlated with age, comorbidities, medications, physical activity, performance, nutrition and handgrip strength. Two genetic variants were significantly associated with handgrip strength, and 12 polymorphisms previously linked to sarcopenia were replicated in relationship to LMI. Methylation in immune‐related genes was enriched for LMI, whereas shorter telomeres, associated with frailty and reduced function, were correlated with lifestyle‐related gene methylation. These findings highlight genetic and epigenetic biomarkers for early diagnosis and treatment.

## AUTHOR CONTRIBUTIONS

Conceptualization: Valentina Ginevičienė, Ildus I. Ahmetov, Vidmantas Alekna and Erinija Pranckevičienė. Methodology: Justina Kilaitė, Valentina Ginevičienė, Alina Urnikytė, Kristijona Gutauskaitė and Laura Jurkūnaitė. Software: Erinija Pranckevičienė, Alina Urnikytė, Valentina Ginevičienė, Kristijona Gutauskaitė, Laura Jurkūnaitė and Justina Kilaitė. Validation: Vidmantas Alekna, Ildus I. Ahmetov, Valentina Ginevičienė, Justina Kilaitė, Erinija Pranckevičienė and Rūta Dadelienė. Formal analysis: Justina Kilaitė, Valentina Ginevičienė, Erinija Pranckevičienė, Kristijona Gutauskaitė, Laura Jurkūnaitė and Rūta Dadelienė. Investigation: Asta Mastavičiūtė, Justina Kilaitė, Rūta Dadelienė, Ieva Eglė Jamontaitė, Alina Urnikytė, Erinija Pranckevičienė and Valentina Ginevičienė. Resources: Valentina Ginevičienė, Alina Urnikytė, Erinija Pranckevičienė and Justina Kilaitė. Data curation: Valentina Ginevičienė, Alina Urnikytė, Erinija Pranckevičienė and Justina Kilaitė. Writing—original draft preparation: Valentina Ginevičienė, Justina Kilaitė, Erinija Pranckevičienė, Laura Jurkūnaitė, Kristijona Gutauskaitė, Alina Urnikytė, Ieva Eglė Jamontaitė and Ildus I. Ahmetov. Writing—review and editing: Valentina Ginevičienė, Ildus I. Ahmetov, Justina Kilaitė, Erinija Pranckevičienė, Asta Mastavičiūtė, Laura Jurkūnaitė and Kristijona Gutauskaitė. Visualization: Valentina Ginevičienė, Justina Kilaitė, Erinija Pranckevičienė, Asta Mastavičiūtė Supervision: Vidmantas Alekna, Valentina Ginevičienė and Ildus I. Ahmetov. Project administration: Vidmantas Alekna. Funding acquisition: Vidmantas Alekna. All authors have read and agreed to the published version of the manuscript and agree to be accountable for all aspects of the work in ensuring that questions related to the accuracy or integrity of any part of the work are appropriately investigated and resolved. All persons designated as authors qualify for authorship, and all those who qualify for authorship are listed.

## CONFLICT OF INTEREST

None declared.

## Supporting information



Supporting Information

## Data Availability

The genotyping raw data, along with GWAS and EWAS summary statistics supporting the findings of this study, are openly available in Figshare, respectively: dx.doi.org/10.6084/m9.figshare.28845761 and dx.doi.org/10.6084/m9.figshare.28874378.

## References

[eph70054-bib-0001] Ainsworth, N. J. , Brender, R. , Gotlieb, N. , Zhao, H. , Blumberger, D. M. , Karp, J. F. , Lenze, E. J. , Nicol, G. E. , Reynolds, C. F. , Wang, W. , & Mulsant, B. H. (2023). Association between lean muscle mass and treatment‐resistant late‐life depression in the IRL‐GRey randomized controlled trial. International Psychogeriatrics, 35(12), 707–716. 36594430 10.1017/S1041610222000862

[eph70054-bib-0002] Atkins, J. L. , Jylhävä, J. , Pedersen, N. L. , Magnusson, P. K. , Lu, Y. , Wang, Y. , Hägg, S. , Melzer, D. , Williams, D. M. , & Pilling, L. C. (2021). A genome‐wide association study of the frailty index highlights brain pathways in ageing. Aging Cell, 20(9), e13459. 34431594 10.1111/acel.13459 PMC8441299

[eph70054-bib-0003] Barazzoni, R. , Jensen, G. L. , Correia, M. I. T. D. , Gonzalez, M. C. , Higashiguchi, T. , Shi, H. P. , Bischoff, S. C. , Boirie, Y. , Carrasco, F. , Cruz‐Jentoft, A. , Fuchs‐Tarlovsky, V. , Fukushima, R. , Heymsfield, S. , Mourtzakis, M. , Muscaritoli, M. , Norman, K. , Nyulasi, I. , Pisprasert, V. , Prado, C. , … Compher, C. (2022). Guidance for assessment of the muscle mass phenotypic criterion for the Global Leadership Initiative on Malnutrition (GLIM) diagnosis of malnutrition. Clinical Nutrition, 41(6), 1425–1433. 35450768 10.1016/j.clnu.2022.02.001

[eph70054-bib-0004] Barbera, M. C. , Guarrera, L. , Re Cecconi, A. D. , Cassanmagnago, G. A. , Vallerga, A. , Lunardi, M. , Checchi, F. , Di Rito, L. , Romeo, M. , Mapelli, S. N. , Schoser, B. , Generozov, E. V. , Jansen, R. , de Geus, E. J. C. , Penninx, B. , van Dongen, J. , Craparotta, I. , Piccirillo, R. , … Bolis, M. , Molecular Genetics Group . (2025). Increased ectodysplasin‐A2‐receptor EDA2R is a ubiquitous hallmark of aging and mediates parainflammatory responses. Nature Communications, 16(1), 1898. 10.1038/s41467-025-56918-3 PMC11847917 39988718

[eph70054-bib-0005] Baumgartner, N. W. , & Kao, S.‐C. (2024). Size or Strength? How components of muscle relate to behavioral and neuroelectric measures of executive function independent of aerobic fitness. Brain and Cognition, 175, 106139. 38364518 10.1016/j.bandc.2024.106139

[eph70054-bib-0005a] Browning, B. L. , Tian, X. , Zhou, Y. , & Browning, S. R. (2021). Fast two‐stage phasing of large‐scale sequence data. American Journal of Human Genetics, 108(10), 1880–1890. 34478634 10.1016/j.ajhg.2021.08.005 PMC8551421

[eph70054-bib-0006] Cawthon, P. M. , Travison, T. G. , Manini, T. M. , Patel, S. , Pencina, K. M. , Fielding, R. A. , Magaziner, J. M. , Newman, A. B. , Brown, T. , Kiel, D. P. , Cummings, S. R. , Shardell, M. , Guralnik, J. M. , Woodhouse, L. J. , Pahor, M. , Binder, E. , D'Agostino, R. B. , Quian‐Li, X. , Orwoll, E. , … Bhasin, S. (2020). Establishing the link between lean mass and grip strength cut points with mobility disability and other health outcomes: Proceedings of the sarcopenia definition and outcomes consortium conference. The Journals of Gerontology‐Series A, Biological Sciences and Medical Sciences, 75(7), 1317–1323. 30869772 10.1093/gerona/glz081 PMC7447857

[eph70054-bib-0007] Cawthon, R. M. (2002). Telomere measurement by quantitative PCR. Nucleic Acids Research, 30(10), e47. 12000852 10.1093/nar/30.10.e47 PMC115301

[eph70054-bib-0008] Cederholm, T. (2015). Overlaps between frailty and sarcopenia definitions. In R. A. Fielding , C. Sieber , & B. Vellas (Eds.), Frailty: Pathophysiology, phenotype and patient care: 83rd Nestlé Nutrition Institute Workshop (Vol. 83, p. 0, March 2014). S. Karger AG. 10.1159/000382063 26484770

[eph70054-bib-0009] Cerezo, M. , Sollis, E. , Ji, Y. , Lewis, E. , Abid, A. , Bircan, K. O. , Hall, P. , Hayhurst, J. , John, S. , Mosaku, A. , Ramachandran, S. , Foreman, A. , Ibrahim, A. , McLaughlin, J. , Pendlington, Z. , Stefancsik, R. , Lambert, S. A. , McMahon, A. , Morales, J. , … Harris, L. W. (2025). The NHGRI‐EBI GWAS catalog: Standards for reusability, sustainability and diversity. Nucleic Acids Research, 53(D1), D998–D1005. 39530240 10.1093/nar/gkae1070 PMC11701593

[eph70054-bib-0010] Craighead, D. H. , Freeberg, K. A. , Heinbockel, T. C. , Rossman, M. J. , Jackman, R. A. , MCcarty, N. P. , Jankowski, L. R. , Nemkov, T. , Reisz, J. A. , D'Alessandro, A. , Chonchol, M. , Bailey, E. F. , & Seals, D. R. (2024). Time‐efficient, high‐resistance inspiratory muscle strength training increases exercise tolerance in midlife and older adults. Medicine & Science in Sports & Exercise, 56(2), https://journals.lww.com/acsm‐msse/fulltext/2024/02000/time_efficient,_high_resistance_inspiratory_muscle.11.aspx 10.1249/MSS.0000000000003291 PMC10840713 37707508

[eph70054-bib-0011] Cruz‐Jentoft, A. J. , Bahat, G. , Bauer, J. , Boirie, Y. , Bruyère, O. , Cederholm, T. , Cooper, C. , Landi, F. , Rolland, Y. , Sayer, A. A. , Schneider, S. M. , Sieber, C. C. , Topinkova, E. , Vandewoude, M. , Visser, M. , & Zamboni, M. , & Writing Group for the European Working Group on Sarcopenia in Older People 2 (EWGSOP2), and the Extended Group for EWGSOP2 . (2019). Sarcopenia: Revised European consensus on definition and diagnosis. Age and Ageing, 48(1), 16–31. 30312372 10.1093/ageing/afy169 PMC6322506

[eph70054-bib-0012] da Silva, S. M. A. , Tempaku, P. F. , Piovezan, R. D. , Andersen, M. L. , Tufik, S. , & D'Almeida, V. (2025). Genetic determinants of muscle health: A population‐based study. The Journal of Frailty & Aging, 14(2), 100013. 40056411 10.1016/j.tjfa.2025.100013 PMC12183942

[eph70054-bib-0013] Fried, L. P. , Tangen, C. M. , Walston, J. , Newman, A. B. , Hirsch, C. , Gottdiener, J. , Seeman, T. , Tracy, R. , Kop, W. J. , Burke, G. , & McBurnie, M. A. (2001). Frailty in older adults: evidence for a phenotype. The Journals of Gerontology: Series A, 56(3), M146–M157. 10.1093/gerona/56.3.m146 11253156

[eph70054-bib-0014] Ginevičienė, V. , Pranckevičienė, E. , Kilaitė, J. , Mastavičiūtė, A. , Dadelienė, R. , Jamontaitė, I. E. , Letukienė, A. , Ahmetov, I. I. , & Alekna, V. (2024). Bibliometric and scientometric analysis on biomarkers and molecular mechanisms for physical frailty and sarcopenia. Frontiers in Medicine, 11, https://www.frontiersin.org/journals/medicine/articles/10.3389/fmed.2024.1326764 10.3389/fmed.2024.1326764 PMC10875138 38375321

[eph70054-bib-0015] Graham, Z. A. , Lavin, K. M. , O'Bryan, S. M. , Thalacker‐Mercer, A. E. , Buford, T. W. , Ford, K. M. , Broderick, T. J. , & Bamman, M. M. (2021). Mechanisms of exercise as a preventative measure to muscle wasting. American Journal of Physiology‐Cell Physiology, 321(1), C40–C57. 33950699 10.1152/ajpcell.00056.2021 PMC8424676

[eph70054-bib-0016] Guilherme, J. P. L. F. , Semenova, E. A. , Borisov, O. V. , Larin, A. K. , Moreland, E. , Generozov, E. V. , & Ahmetov, I. I. (2022). Genomic predictors of testosterone levels are associated with muscle fiber size and strength. European Journal of Applied Physiology, 122(2), 415–423. 34792618 10.1007/s00421-021-04851-w PMC8783862

[eph70054-bib-0017] Guilherme, J. P. L. F. , Semenova, E. A. , Kikuchi, N. , Homma, H. , Kozuma, A. , Saito, M. , Zempo, H. , Matsumoto, S. , Kobatake, N. , Nakazato, K. , Okamoto, T. , John, G. , Yusupov, R. A. , Larin, A. K. , Kulemin, N. A. , Gazizov, I. M. , Generozov, E. V. , & Ahmetov, I. I. (2024). Identification of genomic predictors of muscle fiber size. Cells, 13(14), 1212. 39056794 10.3390/cells13141212 PMC11274365

[eph70054-bib-0018] Haghshenas, S. , Bhai, P. , Aref‐Eshghi, E. , & Sadikovic, B. (2020). Diagnostic utility of genome‐wide DNA methylation analysis in mendelian neurodevelopmental disorders. International Journal of Molecular Sciences, 21(23), 9303. 33291301 10.3390/ijms21239303 PMC7730976

[eph70054-bib-0019] Ji, S. , Qian, Z. , Hu, P. , & Chen, F. (2023). Age‐dependent Changes in skeletal muscle mass and visceral fat area in a Chinese population. Current Medical Science, 43(4), 838–844. 37326887 10.1007/s11596-023-2742-5

[eph70054-bib-0019a] Joglekar, M. V. , Satoor, S. N. , Wong, W. K. M. , Cheng, F. , Ma, R. C. W. , & Hardikar, A. A. (2020). An optimised step‐by‐step protocol for measuring relative telomere length. Methods and Protocols, 3(2), 27. 32260112 10.3390/mps3020027 PMC7359711

[eph70054-bib-0020] Jones, G. , Pilling, L. C. , Kuo, C.‐L. , Kuchel, G. , Ferrucci, L. , & Melzer, D. (2020). Sarcopenia and variation in the human leukocyte antigen complex. The Journals of Gerontology: Series A, 75(2), 301–308. 10.1093/gerona/glz042 PMC7176057 30772894

[eph70054-bib-0021] Jones, G. , Trajanoska, K. , Santanasto, A. J. , Stringa, N. , Kuo, C.‐L. , Atkins, J. L. , Lewis, J. R. , Duong, T. , Hong, S. , Biggs, M. L. , Luan, J. , Sarnowski, C. , Lunetta, K. L. , Tanaka, T. , Wojczynski, M. K. , Cvejkus, R. , Nethander, M. , Ghasemi, S. , Yang, J. , … Pilling, L. C. (2021). Genome‐wide meta‐analysis of muscle weakness identifies 15 susceptibility loci in older men and women. Nature Communications, 12(1), 654. 10.1038/s41467-021-20918-w PMC7844411 33510174

[eph70054-bib-0022] Kameda, M. , Teruya, T. , Yanagida, M. , & Kondoh, H. (2020). Frailty markers comprise blood metabolites involved in antioxidation, cognition, and mobility. Proceedings of the National Academy of Sciences, 117(17), 9483–9489. 10.1073/pnas.1920795117 PMC7196897 32295884

[eph70054-bib-0023] Kinoshita, K. , Matsui, Y. , Hirano, Y. , Satake, S. , Osuka, Y. , Li, J. , Yoshiura, K. , Hori, N. , & Arai, H. (2025). Association between the presence or absence of muscle mass assessment in sarcopenia diagnosis and poor health outcomes: A follow‐up study of older outpatients at a frailty clinic. Geriatrics & Gerontology International, 25(4), 553–559. 40051215 10.1111/ggi.70020

[eph70054-bib-0024] Kitamura, M. , Izawa, K. P. , Ishihara, K. , Matsuda, H. , Okamura, S. , & Fujioka, K. (2021). Physical activity and sarcopenia in community‐dwelling older adults with long‐term care insurance. European Journal of Investigation in Health, Psychology and Education, 11(4), 1610–1618. 34940392 10.3390/ejihpe11040114 PMC8700727

[eph70054-bib-0025] Kmiołek, T. , Filipowicz, G. , Bogucka, D. , Wajda, A. , Ejma‐Multański, A. , Stypińska, B. , Modzelewska, E. , Kaliberda, Y. , Radkowski, M. , Targowski, T. , Wrona, J. , & Paradowska‐Gorycka, A. (2023). Aging and the impact of global DNA methylation, telomere shortening, and total oxidative status on sarcopenia and frailty syndrome. Immunity & Ageing, 20(1), 61. 37964387 10.1186/s12979-023-00384-2 PMC10644469

[eph70054-bib-0026] Kolberg, L. , Raudvere, U. , Kuzmin, I. , Vilo, J. , & Peterson, H. (2020). Gprofiler2—An R package for gene list functional enrichment analysis and namespace conversion toolset g:Profiler [version 2; peer review: 2 approved]. F1000Research, 9(ELIXIR), 709. 10.12688/f1000research.24956.1 PMC7859841 33564394

[eph70054-bib-0027] Li, Z. , Tong, X. , Ma, Y. , Bao, T. , & Yue, J. (2022). Prevalence of depression in patients with sarcopenia and correlation between the two diseases: Systematic review and meta‐analysis. Journal of Cachexia, Sarcopenia and Muscle, 13(1), 128–144. 34997702 10.1002/jcsm.12908 PMC8818614

[eph70054-bib-0027a] Loh, P. R. , Danecek, P. , Palamara, P. F. , Fuchsberger, C. , Reshef, Y. A. , Finucane, H. K. , Schoenherr, S. , Forer, L. , McCarthy, S. , Abecasis, G. R. , Durbin, R. , & Price, A. L. (2016). Reference‐based phasing using the Haplotype Reference Consortium panel. Nature Genetics, 48(11), 1443–1448. 27694958 10.1038/ng.3679 PMC5096458

[eph70054-bib-0028] Lu, Y. , Wang, Y. , & Lu, Q. (2019). Effects of exercise on muscle fitness in dialysis patients: A systematic review and meta‐analysis. American Journal of Nephrology, 50(4), 291–302. 31480056 10.1159/000502635

[eph70054-bib-0029] Maciejewska‐Skrendo, A. , Sawczuk, M. , Cięszczyk, P. , & Ahmetov, I. I. (2019). Genes and power athlete status. In D. Barh & I. Ahmetov (Eds.), Sports, Exercise, and Nutritional Genomics: Current Status and Future Directions (pp. 41–72). Academic Press, 10.1016/B978-0-12-816193-7.00003-8

[eph70054-bib-0030] Mak, J. K. L. , Qin, C. , Krüger, M. , Kuukka, A. , FinnGen , Hägg, S. , Lin, J. , & Jylhävä, J. (2025). Large‐scale genome‐wide analyses with proteomics integration reveal novel loci and biological insights into frailty. Nature Aging, 5(8), 1589–1600. 40764432 10.1038/s43587-025-00925-y PMC12350161

[eph70054-bib-0028a] Marchini, J. , Howie, B. , Myers, S. , McVean, G. , & Donnelly, P. (2007). A new multipoint method for genome‐wide association studies by imputation of genotypes. Nature Genetics, 39, 906–913. 17572673 10.1038/ng2088

[eph70054-bib-0031] Mekli, K. , Stevens, A. , Marshall, A. D. , Arpawong, T. E. , Phillips, D. F. , Tampubolon, G. , Lee, J. , Prescott, C. A. , Nazroo, J. Y. , & Pendleton, N. (2018). Frailty Index associates with GRIN2B in two representative samples from the United States and the United Kingdom. PLoS ONE, 13(11), e0207824. 30475886 10.1371/journal.pone.0207824 PMC6258126

[eph70054-bib-0032] Moreland, E. , Borisov, O. V. , Semenova, E. A. , Larin, A. K. , Andryushchenko, O. N. , Andryushchenko, L. B. , Generozov, E. V. , Williams, A. G. , & Ahmetov, I. I. (2022). Polygenic profile of elite strength athletes. Journal of Strength and Conditioning Research, 36(9), 2509–2514. 33278272 10.1519/JSC.0000000000003901

[eph70054-bib-0033] Oudbier, S. J. , Goh, J. , Looijaard, S. M. L. M. , Reijnierse, E. M. , Meskers, C. G. M. , & Maier, A. B. (2022). Pathophysiological mechanisms explaining the association between low skeletal muscle mass and cognitive function. The Journals of Gerontology: Series A, 77(10), 1959–1968. 10.1093/gerona/glac121 PMC9536455 35661882

[eph70054-bib-0034] Pei, Y. F. , Liu, Y. Z. , Yang, X. L. , Zhang, H. , Feng, G. J. , Wei, X. T. , & Zhang, L. (2020). The genetic architecture of appendicular lean mass characterized by association analysis in the UK Biobank study. Communications Biology, 3(1), 608. 33097823 10.1038/s42003-020-01334-0 PMC7585446

[eph70054-bib-0035] Peters, T. J. , Meyer, B. , Ryan, L. , Achinger‐Kawecka, J. , Song, J. , Campbell, E. M. , Qu, W. , Nair, S. , Loi‐Luu, P. , Stricker, P. , Lim, E. , Stirzaker, C. , Clark, S. J. , & Pidsley, R. (2024). Characterisation and reproducibility of the HumanMethylationEPIC v2.0 BeadChip for DNA methylation profiling. BioMed Central Genomics [Electronic Resource], 25(1), 251. 38448820 10.1186/s12864-024-10027-5 PMC10916044

[eph70054-bib-0036] Riemondy, K. A. , Sheridan, R. M. , Gillen, A. , Yu, Y. , Bennett, C. G. , & Hesselberth, J. R. (2017). valr: Reproducible genome interval arithmetic in R. F1000Research, 6, 1025. 28751969 10.12688/f1000research.11997.1 PMC5506536

[eph70054-bib-0037] Roberts, H. C. , Denison, H. J. , Martin, H. J. , Patel, H. P. , Syddall, H. , Cooper, C. , & Sayer, A. A. (2011). A review of the measurement of grip strength in clinical and epidemiological studies: Towards a standardised approach. Age and Ageing, 40(4), 423–429. 21624928 10.1093/ageing/afr051

[eph70054-bib-0038] Rubenstein, L. Z. , Harker, J. O. , Salvà, A. , Guigoz, Y. , & Vellas, B. (2001). Screening for undernutrition in geriatric practice: Developing the short‐form mini‐nutritional assessment (MNA‐SF). The Journals of Gerontology: Series A, 56(6), M366–M372. 10.1093/gerona/56.6.m366 11382797

[eph70054-bib-0039] Semenova, E. A. , Pranckevičienė, E. , Bondareva, E. A. , Gabdrakhmanova, L. J. , & Ahmetov, I. I. (2023). Identification and characterization of genomic predictors of sarcopenia and sarcopenic obesity using UK biobank data. Nutrients, 15(3), 758. 36771461 10.3390/nu15030758 PMC9920138

[eph70054-bib-0040] Shi, L. , Zhang, L. , Zhang, D. , & Chen, Z. (2023). Association between systemic immune‐inflammation index and low muscle mass in US adults: A cross‐sectional study. BioMed Central Public Health [Electronic Resource], 23(1), 1416. 37488531 10.1186/s12889-023-16338-8 PMC10367418

[eph70054-bib-0041] Singmann, P. , Shem‐Tov, D. , Wahl, S. , Grallert, H. , Fiorito, G. , Shin, S.‐Y. , Schramm, K. , Wolf, P. , Kunze, S. , Baran, Y. , Guarrera, S. , Vineis, P. , Krogh, V. , Panico, S. , Tumino, R. , Kretschmer, A. , Gieger, C. , Peters, A. , Prokisch, H. , … Halperin, E. (2015). Characterization of whole‐genome autosomal differences of DNA methylation between men and women. Epigenetics & Chromatin, 8(1), 43. 26500701 10.1186/s13072-015-0035-3 PMC4615866

[eph70054-bib-0042] Sirago, G. , Pellegrino, M. A. , Bottinelli, R. , Franchi, M. V. , & Narici, M. V. (2023). Loss of neuromuscular junction integrity and muscle atrophy in skeletal muscle disuse. Ageing Research Reviews, 83, 101810. 36471545 10.1016/j.arr.2022.101810

[eph70054-bib-0043] Tessier, A.‐J. , Wing, S. S. , Rahme, E. , Morais, J. A. , & Chevalier, S. (2022). Association of low muscle mass with cognitive function during a 3‐year follow‐up among adults aged 65 to 86 years in the Canadian Longitudinal Study on Aging. Journal of the American Medical Association Network Open, 5(7), e2219926. 10.1001/jamanetworkopen.2022.19926 PMC9250053 35796211

[eph70054-bib-0044] Tikkanen, E. , Gustafsson, S. , Amar, D. , Shcherbina, A. , Waggott, D. , Ashley, E. A. , & Ingelsson, E. (2018). Biological insights into muscular strength: Genetic findings in the UK biobank. Scientific Reports, 8(1), 6451. 29691431 10.1038/s41598-018-24735-y PMC5915424

[eph70054-bib-0045] Timmins, I. R. , Zaccardi, F. , Nelson, C. P. , Franks, P. W. , Yates, T. , & Dudbridge, F. (2020). Genome‐wide association study of self‐reported walking pace suggests beneficial effects of brisk walking on health and survival. Communications Biology, 3(1), 634. 33128006 10.1038/s42003-020-01357-7 PMC7599247

[eph70054-bib-0046] Veronese, N. , Smith, L. , Cereda, E. , Maggi, S. , Barbagallo, M. , Dominguez, L. J. , & Koyanagi, A. (2021). Multimorbidity increases the risk for sarcopenia onset: Longitudinal analyses from the English Longitudinal Study of Ageing. Experimental Gerontology, 156, 111624. 34767942 10.1016/j.exger.2021.111624

[eph70054-bib-0047] Villicana, S. , Castillo‐Fernandez, J. , Hannon, E. , Christiansen, C. , Tsai, P. C. , Maddock, J. , Kuh, D. , Suderman, M. , Power, C. , Relton, C. , , Ploubidis, G. , Wong, A. , Hardy, R. , Goodman, A. , Ong, K. K. , & Bell, J. T. (2023). Genetic impacts on DNA methylation help elucidate regulatory genomic processes. Genome Biology, 24(1), 176. 37525248 10.1186/s13059-023-03011-x PMC10391992

[eph70054-bib-0048] Wang, H. , Huang, W. Y. , & Zhao, Y. (2022). Efficacy of exercise on muscle function and physical performance in older adults with sarcopenia: An updated systematic review and meta‐analysis. International Journal of Environmental Research and Public Health, 19(13), 8212. 35805870 10.3390/ijerph19138212 PMC9266336

[eph70054-bib-0049] Wu, S. , & Peng, L. (2024). Increased CCT5 expression is a potential unfavourable factor promoting the growth of nasopharyngeal carcinoma. Journal of International Medical Research, 52(9), 03000605241271754. 39286844 10.1177/03000605241271754 PMC11409311

[eph70054-bib-0050] Yamada, M. , Moriguch, Y. , Mitani, T. , Aoyama, T. , & Arai, H. (2014). Age‐dependent changes in skeletal muscle mass and visceral fat area in Japanese adults from 40 to 79 years‐of‐age. Geriatrics & Gerontology International, 14(S1), 8–14. 10.1111/ggi.12209 24450556

[eph70054-bib-0051] Yang, F. , Zhang, X. , Dai, W. , Xu, K. , Mei, Y. , Liu, T. , Wang, K. , Liang, Q. , Guo, P. , Liang, C. , & Meng, J. (2025). Multivariate genome‐wide analysis of sarcopenia reveals genetic comorbidity with urological diseases. Experimental Gerontology, 206, 112783. 40378933 10.1016/j.exger.2025.112783

[eph70054-bib-0052] Ye, Y. , Noche, R. B. , Szejko, N. , Both, C. P. , Acosta, J. N. , Leasure, A. C. , Brown, S. C. , Sheth, K. N. , Gill, T. M. , Zhao, H. , & Falcone, G. J. (2023). A genome‐wide association study of frailty identifies significant genetic correlation with neuropsychiatric, cardiovascular, and inflammation pathways. GeroScience, 45(4), 2511–2523. 36928559 10.1007/s11357-023-00771-z PMC10651618

[eph70054-bib-0053] Zempo, H. , Miyamoto‐Mikami, E. , Kikuchi, N. , Fuku, N. , Miyachi, M. , & Murakami, H. (2017). Heritability estimates of muscle strength‐related phenotypes: A systematic review and meta‐analysis. Scandinavian Journal of Medicine & Science in Sports, 27(12), 1537–1546. 27882617 10.1111/sms.12804

[eph70054-bib-0054] Zhou, W. , Triche, T. J. , Laird, P. W. Jr , & Shen, H. (2018). SeSAMe: Reducing artifactual detection of DNA methylation by Infinium BeadChips in genomic deletions. Nucleic Acids Research, 46(20), e123–e123. 30085201 10.1093/nar/gky691 PMC6237738

