# Supplementary material for: A multi‐omics investigation of sarcopenia and frailty: Integrating genomic, epigenomic and telomere length data
Source: Exp Physiol. 2025 Sep 27:10.1113/EP092853. Online ahead of print. doi: 10.1113/EP092853 (PMC13394718; doi:10.1113/EP092853)
Supplement: Supplementary file 1 — Supporting Information [file EPH-9999-0-s001.docx]

**Supplementary File**

**A multi-omics investigation of sarcopenia and frailty: integrating genomic, epigenomic, and telomere length data**

Valentina Ginevičienė^1^, Erinija Pranckevičienė^1,2^, Alina Urnikytė^3^, Laura Jurkūnaitė^1^, Kristijona Gutauskaitė^1^, Rūta Dadelienė^1^, Justina Kilaitė^1^, Ieva Eglė Jamontaitė^1^, Asta Mastavičiūtė^1^, Ildus I. Ahmetov^1,4^*, Vidmantas Alekna^1^

^1^ Faculty of Medicine, Vilnius University, Vilnius, Lithuania

^2^ Faculty of Informatics, Vytautas Magnus University, Kaunas, Lithuania

^3^ Translational Health Research Institute, Faculty of Medicine, Vilnius University, Vilnius, Lithuania

^4^ Research Institute for Sport and Exercise Sciences, Liverpool John Moores University, Liverpool, United Kingdom

| **Males** | **Females** |
| --- | --- |
| 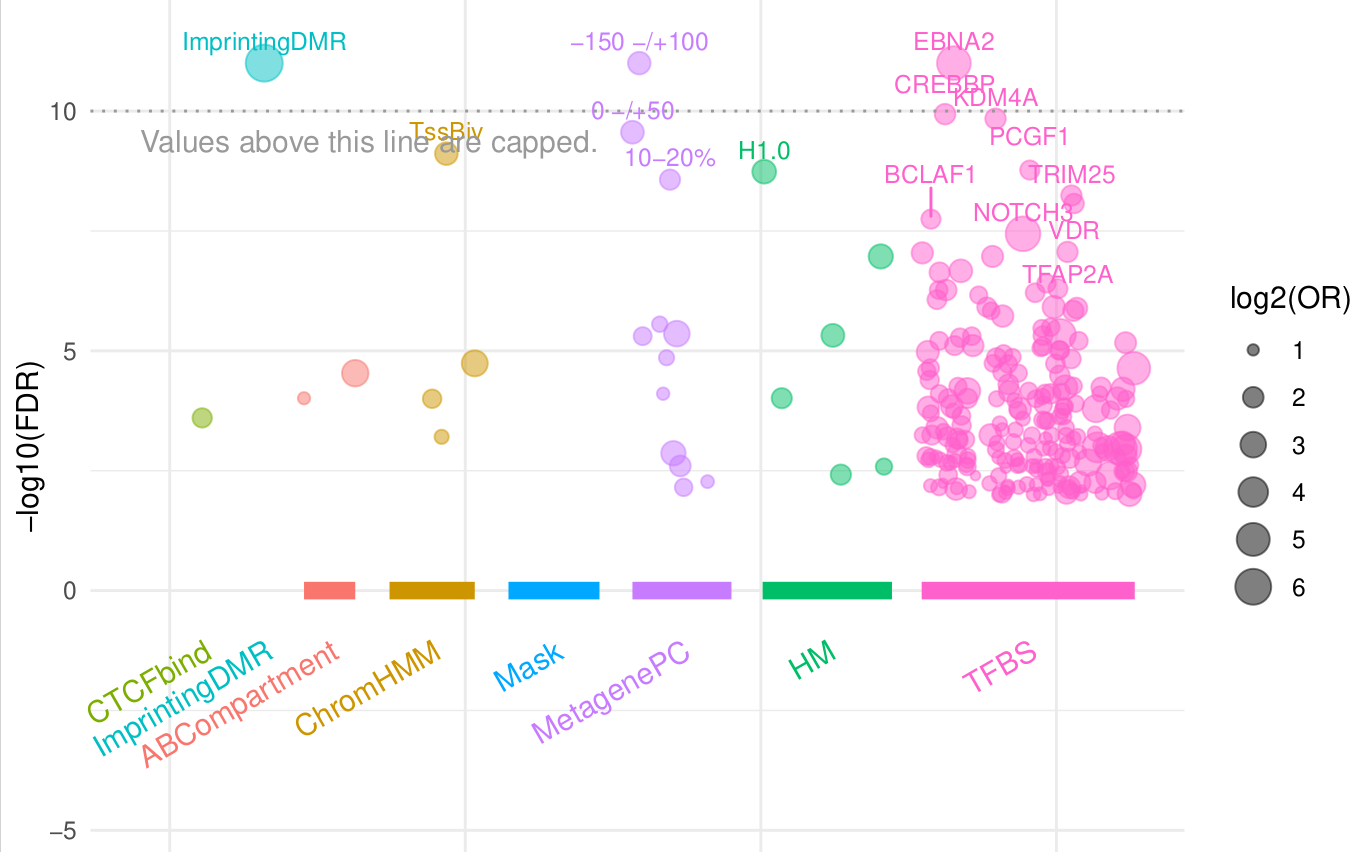 | 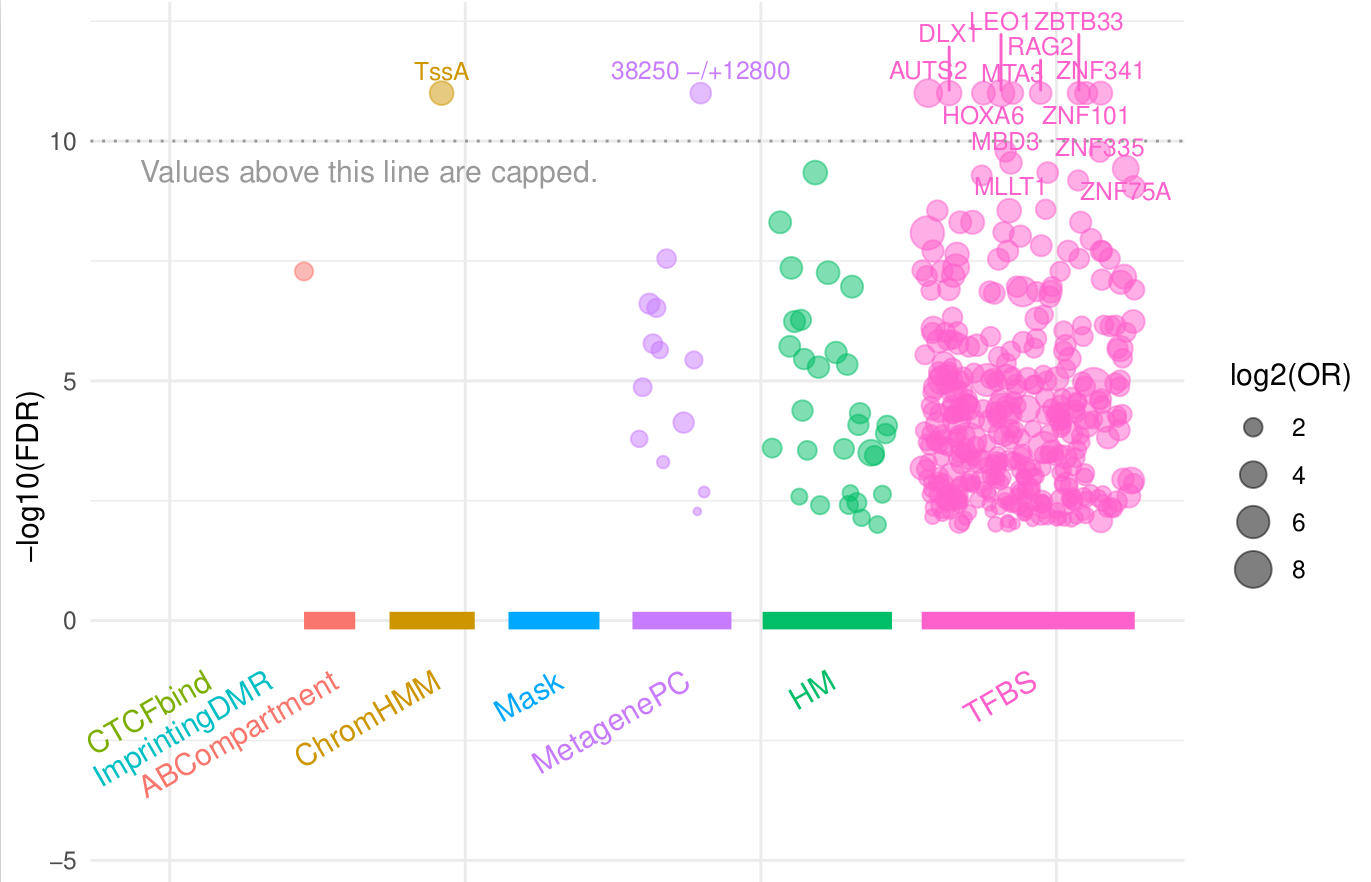 |
|  |  |

**Supplementary Figure 1.** Enrichment of probes significantly associated with lean mass index in different databases of functional genomic elements.

| **Males** | **Females** |
| --- | --- |
| 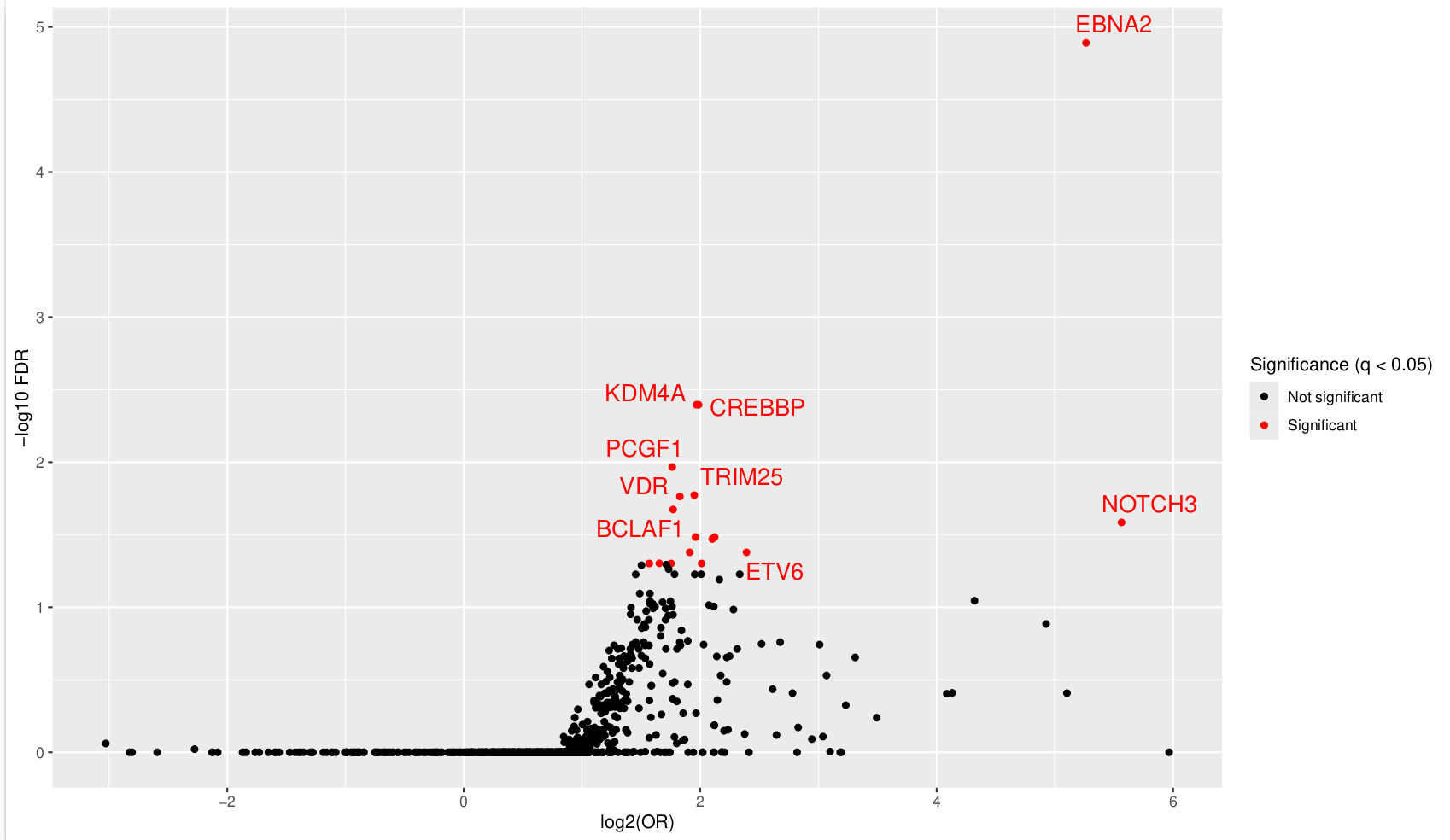 | 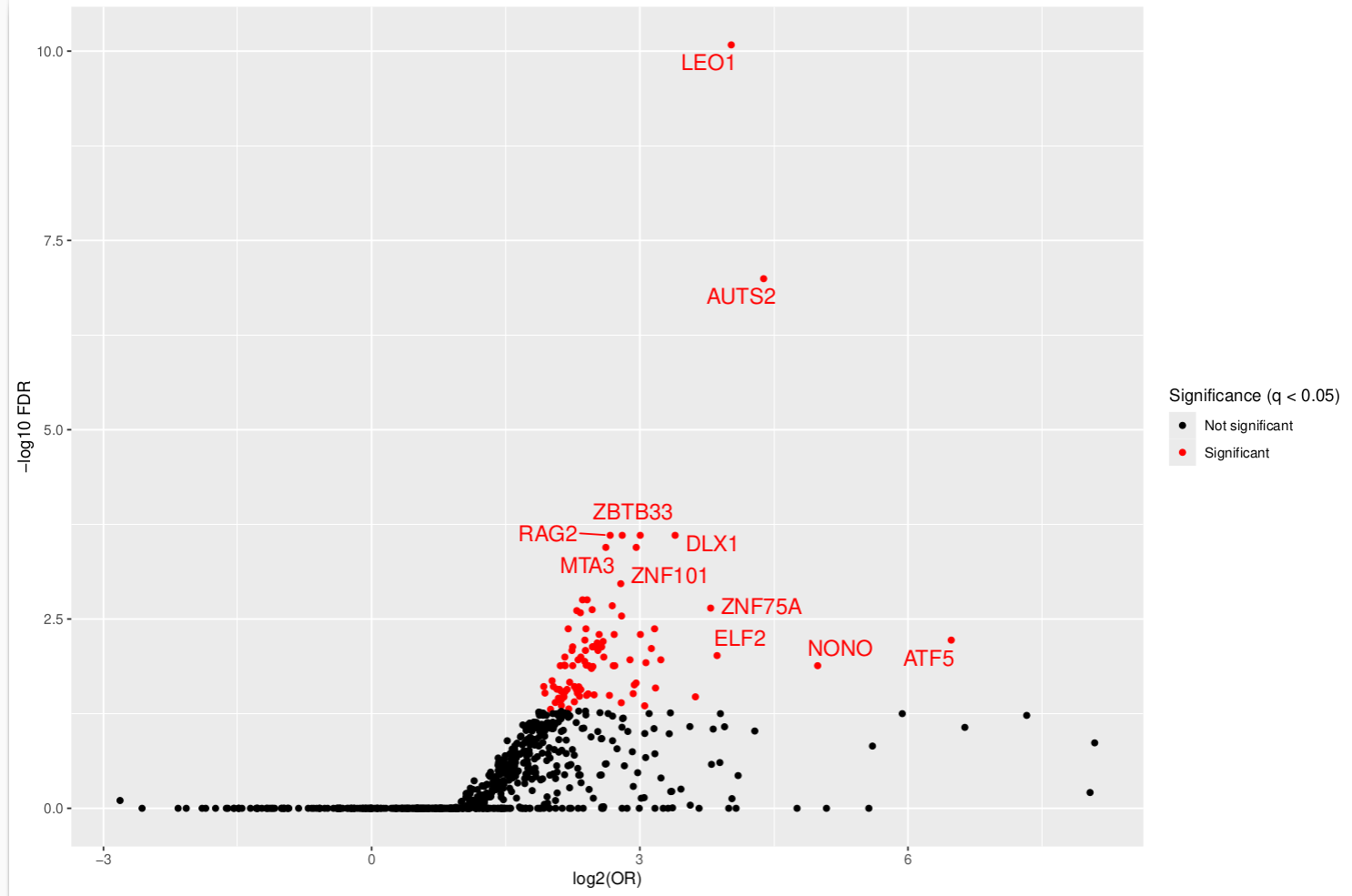 |

**Supplementary Figure 2.** Transcription factors in which binding sites were most significantly enriched by probes whose methylation level is associated with lean mass index.


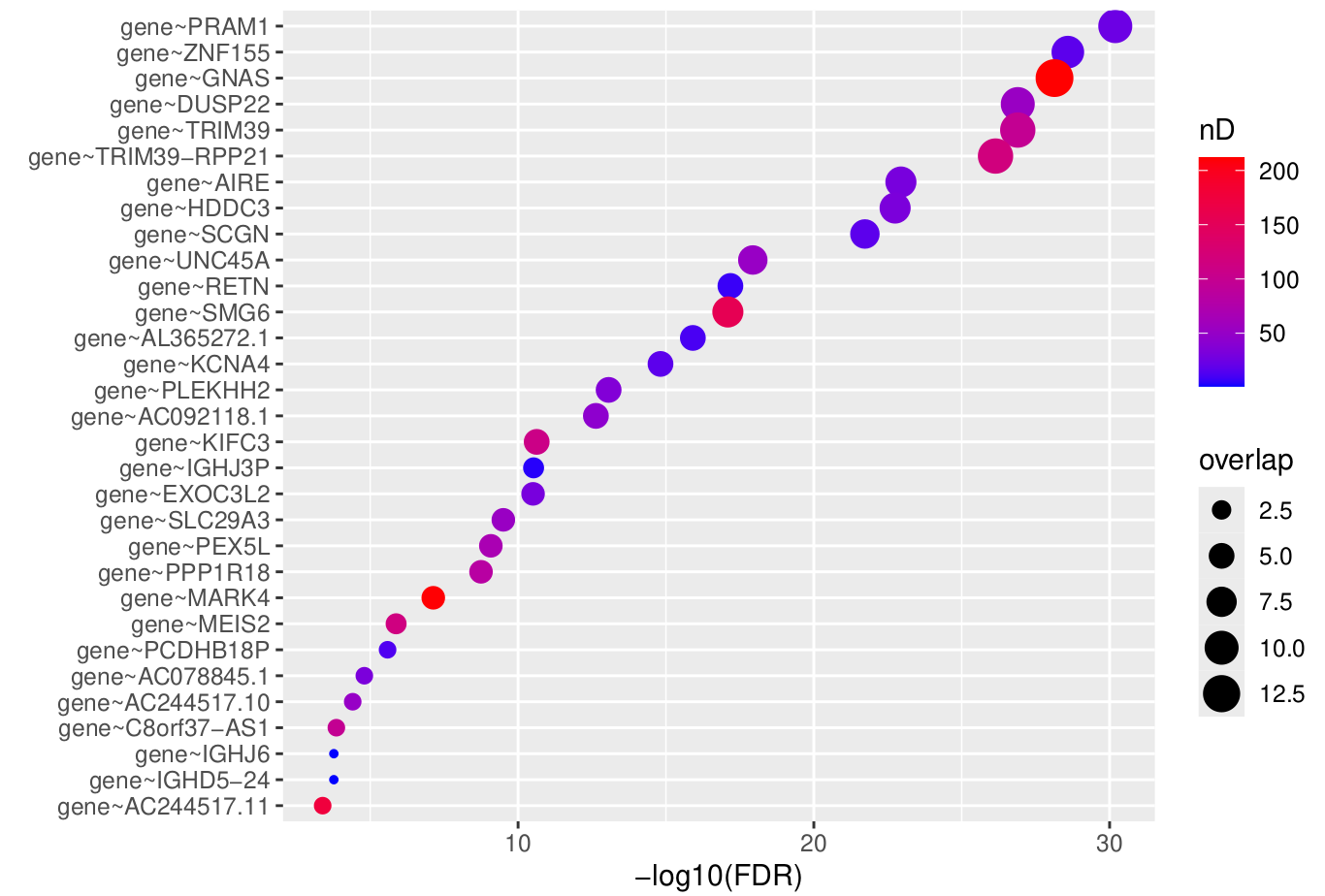


**Supplementary Figure 3.** Genes enriched by probes in males


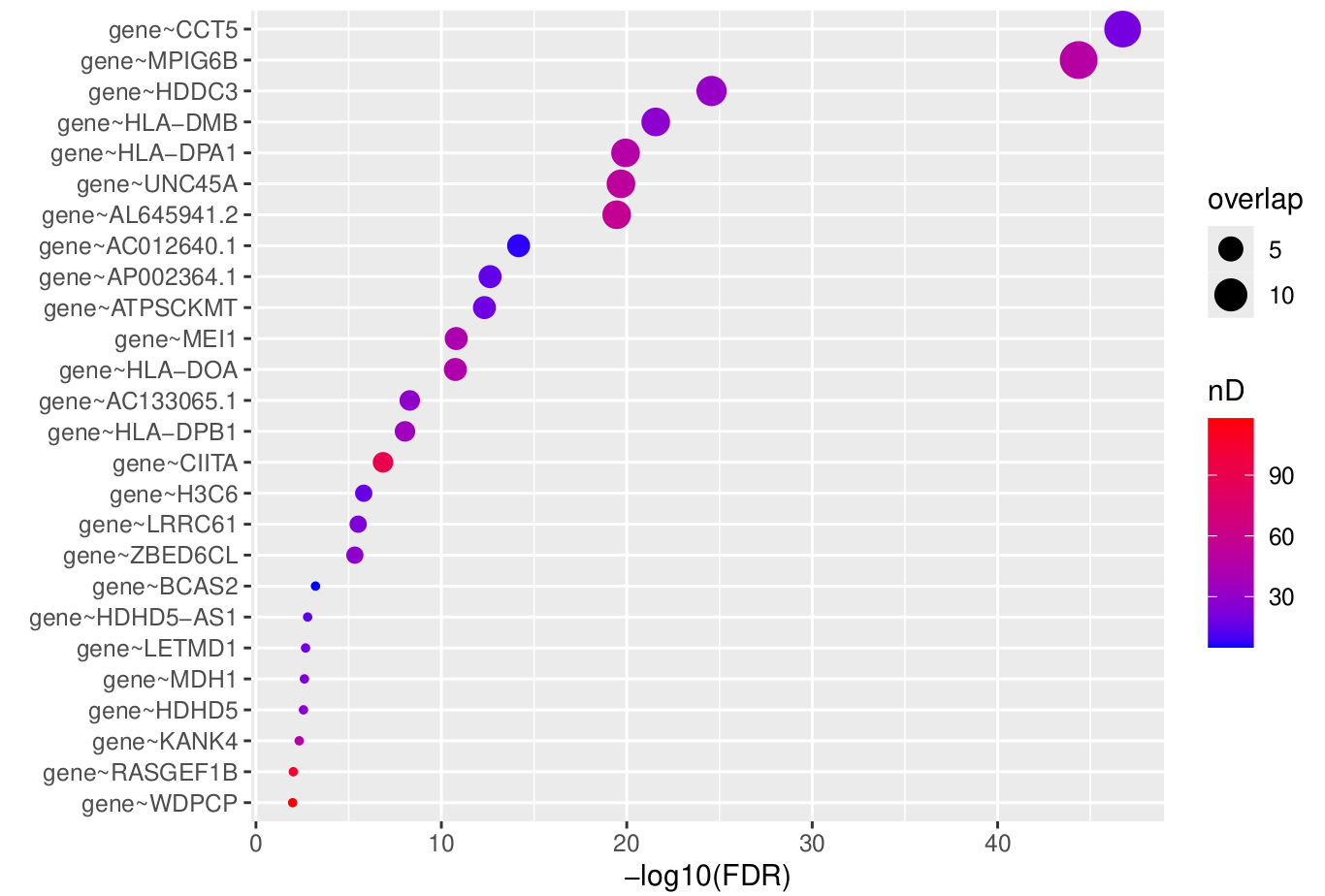


**Supplementary Figure 4.** Genes enriched by probes in females

**Supplementary Table 1.** Enrichment of annotations of male and female genes

| **Term_id** | **source** | **pvalue** | **term_name** |
| --- | --- | --- | --- |
| **Males** |  |  |  |
| CORUM:1985 | CORUM | 0.04 | AIRE homodimer complex |
| **Females** |  |  |  |
| GO:0002503 | GO:BP | 0 | peptide antigen assembly with MHC class II protein complex |
| GO:0002399 | GO:BP | 0 | MHC class II protein complex assembly |
| GO:0002501 | GO:BP | 0 | peptide antigen assembly with MHC protein complex |
| GO:0002396 | GO:BP | 0 | MHC protein complex assembly |
| GO:0019886 | GO:BP | 0 | antigen processing and presentation of exogenous peptide antigen via MHC class II |
| GO:0002495 | GO:BP | 0 | antigen processing and presentation of peptide antigen via MHC class II |
| GO:0002504 | GO:BP | 0 | antigen processing and presentation of peptide or polysaccharide antigen via MHC class II |
| GO:0002478 | GO:BP | 0 | antigen processing and presentation of exogenous peptide antigen |
| GO:0019884 | GO:BP | 0 | antigen processing and presentation of exogenous antigen |
| GO:0048002 | GO:BP | 0 | antigen processing and presentation of peptide antigen |
| GO:0019882 | GO:BP | 0 | antigen processing and presentation |
| GO:0042613 | GO:CC | 0 | MHC class II protein complex |
| GO:0042611 | GO:CC | 0 | MHC protein complex |
| GO:0005770 | GO:CC | 0 | late endosome |
| GO:0031902 | GO:CC | 0 | late endosome membrane |
| GO:0098553 | GO:CC | 0.03 | lumenal side of endoplasmic reticulum membrane |
| GO:0023026 | GO:MF | 0 | MHC class II protein complex binding |
| GO:0023023 | GO:MF | 0 | MHC protein complex binding |
| GO:0042605 | GO:MF | 0 | peptide antigen binding |
| GO:0003823 | GO:MF | 0 | antigen binding |
| GO:0032395 | GO:MF | 0.01 | MHC class II receptor activity |
| GO:0042277 | GO:MF | 0.02 | peptide binding |
| HP:0033557 | HP | 0.03 | Anti-proteinase 3 antibody positivity |
| HP:0034104 | HP | 0.03 | Anti-neutrophil elastase antibody positivity |
| HP:0033559 | HP | 0.05 | Anti-myeloperoxidase antibody positivity |
| HPA:0310443 | HPA | 0 | lymph node; non-germinal center cells[High] |
| HPA:0310433 | HPA | 0.01 | lymph node; germinal center cells[High] |
| HPA:0530721 | HPA | 0.02 | spleen; cells in white pulp[≥Low] |
| HPA:0530723 | HPA | 0.03 | spleen; cells in white pulp[High] |
| KEGG:04612 | KEGG | 0 | Antigen processing and presentation |
| KEGG:05310 | KEGG | 0 | Asthma |
| KEGG:05330 | KEGG | 0 | Allograft rejection |
| KEGG:05145 | KEGG | 0 | Toxoplasmosis |
| KEGG:05332 | KEGG | 0 | Graft-versus-host disease |
| KEGG:04940 | KEGG | 0 | Type I diabetes mellitus |
| KEGG:04672 | KEGG | 0 | Intestinal immune network for IgA production |
| KEGG:05322 | KEGG | 0 | Systemic lupus erythematosus |
| KEGG:05320 | KEGG | 0 | Autoimmune thyroid disease |
| KEGG:05321 | KEGG | 0 | Inflammatory bowel disease |
| KEGG:05164 | KEGG | 0 | Influenza A |
| KEGG:05416 | KEGG | 0 | Viral myocarditis |
| KEGG:05152 | KEGG | 0 | Tuberculosis |
| KEGG:05140 | KEGG | 0 | Leishmaniasis |
| KEGG:05150 | KEGG | 0 | Staphylococcus aureus infection |
| KEGG:05323 | KEGG | 0 | Rheumatoid arthritis |
| KEGG:04658 | KEGG | 0 | Th1 and Th2 cell differentiation |
| KEGG:04640 | KEGG | 0 | Hematopoietic cell lineage |
| KEGG:04659 | KEGG | 0 | Th17 cell differentiation |
| KEGG:04145 | KEGG | 0 | Phagosome |
| KEGG:04514 | KEGG | 0 | Cell adhesion molecules |
| KEGG:05169 | KEGG | 0 | Epstein-Barr virus infection |
| KEGG:05166 | KEGG | 0 | Human T-cell leukemia virus 1 infection |
| REAC:R-HSA-2132295 | REAC | 0 | MHC class II antigen presentation |
| REAC:R-HSA-877300 | REAC | 0.02 | Interferon gamma signaling |
| REAC:R-HSA-202430 | REAC | 0.04 | Translocation of ZAP-70 to Immunological synapse |
| WP:WP2328 | WP | 0 | Allograft rejection |
| WP:WP4217 | WP | 0 | Ebola virus infection in host |

**Supplementary Table 2.** Methylated genes in males enriched by probes associated with lean mass index (FDR< 1*E-2)

| **Gene** | **Gene ID** | **Description** | **Methylation status** | **Summary** |
| --- | --- | --- | --- | --- |
| PRAM1 | 84106 | PML-RARA regulated adaptor molecule 1 | Hyper | The protein encoded by this gene is similar to FYN binding protein (FYB/SLAP-130), an adaptor protein involved in T cell receptor mediated signaling. This gene is expressed and regulated during normal myelopoiesis. The expression of this gene is induced by retinoic acid and is inhibited by the expression of PML-RARalpha, a fusion protein of promyelocytic leukemia (PML) and the retinoic acid receptor-alpha (RARalpha). [provided by RefSeq, Jul 2008] |
| ZNF155 | 7711 | zinc finger protein 155 | Hypo | Predicted to enable DNA-binding transcription factor activity. Predicted to be involved in regulation of DNA-templated transcription. Predicted to be located in nucleus. [provided by Alliance of Genome Resources, Mar 2025] |
| GNAS | 2778 | GNAS complex locus | Hyper | This locus has a highly complex imprinted expression pattern. It gives rise to maternally, paternally, and biallelically expressed transcripts that are derived from four alternative promoters and 5' exons. Some transcripts contain a differentially methylated region (DMR) at their 5' exons, and this DMR is commonly found in imprinted genes and correlates with transcript expression. An antisense transcript is produced from an overlapping locus on the opposite strand. One of the transcripts produced from this locus, and the antisense transcript, are paternally expressed noncoding RNAs, and may regulate imprinting in this region. In addition, one of the transcripts contains a second overlapping ORF, which encodes a structurally unrelated protein - Alex. Alternative splicing of downstream exons is also observed, which results in different forms of the stimulatory G-protein alpha subunit, a key element of the classical signal transduction pathway linking receptor-ligand interactions with the activation of adenylyl cyclase and a variety of cellular reponses. Multiple transcript variants encoding different isoforms have been found for this gene. Mutations in this gene result in pseudohypoparathyroidism type 1a, pseudohypoparathyroidism type 1b, Albright hereditary osteodystrophy, pseudopseudohypoparathyroidism, McCune-Albright syndrome, progressive osseus heteroplasia, polyostotic fibrous dysplasia of bone, and some pituitary tumors. [provided by RefSeq, Aug 2012] |
| DUSP22 | 56940 | dual specificity phosphatase 22 | Hyper | Enables phosphoprotein phosphatase activity; protein tyrosine kinase binding activity; and protein tyrosine kinase inhibitor activity. Involved in several processes, including cellular response to epidermal growth factor stimulus; negative regulation of T cell receptor signaling pathway; and negative regulation of focal adhesion assembly. Acts upstream of or within negative regulation of transcription by RNA polymerase II. Located in cytoplasm; leading edge of lamellipodium; and plasma membrane. Part of filamentous actin. [provided by Alliance of Genome Resources, Mar 2025] |
| TRIM39 | 56658 | tripartite motif containing 39 | Hyper | The protein encoded by this gene is a member of the tripartite motif (TRIM) family. The TRIM motif includes three zinc-binding domains, a RING, a B-box type 1 and a B-box type 2, and a coiled-coil region. The function of this protein has not been identified. This gene lies within the major histocompatibility complex class I region on chromosome 6. Alternate splicing results in two transcript variants encoding different isoforms. [provided by RefSeq, Jul 2008] |
| RPP21 | 79897 | ribonuclease P subunit p21 | Hypo | RPP21 is a protein subunit of nuclear ribonuclease P, which processes the 5-prime leader sequence of precursor tRNAs (Jarrous et al., 2001 [PubMed 11497433]).[supplied by OMIM, Jan 2009] |
| AIRE | 326 | autoimmune regulator | Hyper | This gene encodes a transcriptional regulator that forms nuclear bodies and interacts with the transcriptional coactivator CREB binding protein. The encoded protein plays an important role in immunity by regulating the expression of autoantigens and negative selection of autoreactive T-cells in the thymus. Mutations in this gene cause the rare autosomal-recessive systemic autoimmune disease termed autoimmune polyendocrinopathy with candidiasis and ectodermal dystrophy (APECED). [provided by RefSeq, Jun 2012] |
| HDDC3 | 374659 | HD domain containing 3 | Hypo | Predicted to enable guanosine-3',5'-bis(diphosphate) 3'-diphosphatase activity. [provided by Alliance of Genome Resources, Mar 2025] |
| SCGN | 10590 | secretagogin, EF-hand calcium binding protein | Hypo | The encoded protein is a secreted calcium-binding protein which is found in the cytoplasm. It is related to calbindin D-28K and calretinin. This protein is thought to be involved in KCL-stimulated calcium flux and cell proliferation. [provided by RefSeq, Jul 2008] |
| UNC45A | 55898 | unc-45 myosin chaperone A | Hypo | This gene encodes a regulatory component of the progesterone receptor/heat shock protein 90 chaperoning complex, which functions in the assembly and folding of the progesterone receptor. The encoded protein is thought to be essential for normal cell proliferation, and for the accumulation of myosin during development of muscle cells. [provided by RefSeq, Sep 2018] |

**Supplementary Table 3.** Methylated genes in females enriched by probes associated with lean mass index (FDR< 1*E-2)

| **Gene** | **Gene ID** | **Description** | **Methylation status** | **Summary** |
| --- | --- | --- | --- | --- |
| CCT5 | 22948 | chaperonin containing TCP1 subunit 5 | Hypo | The protein encoded by this gene is a molecular chaperone that is a member of the chaperonin containing TCP1 complex (CCT), also known as the TCP1 ring complex (TRiC). This complex consists of two identical stacked rings, each containing eight different proteins. Unfolded polypeptides enter the central cavity of the complex and are folded in an ATP-dependent manner. The complex folds various proteins, including actin and tubulin. Mutations in this gene cause hereditary sensory and autonomic neuropathy with spastic paraplegia (HSNSP). Alternative splicing results in multiple transcript variants. Related pseudogenes have been identified on chromosomes 5 and 13. [provided by RefSeq, Apr 2015] |
| MPIG6B | 80739 | megakaryocyte and platelet inhibitory receptor G6b | Hyper | This gene is a member of the immunoglobulin (Ig) superfamily and is located in the major histocompatibility complex (MHC) class III region. The protein encoded by this gene is a glycosylated, plasma membrane-bound cell surface receptor, but soluble isoforms encoded by some transcript variants have been found in the endoplasmic reticulum and Golgi before being secreted. Multiple transcript variants encoding different isoforms have been found for this gene. [provided by RefSeq, Jul 2008] |
| HDDC3 | 374659 | HD domain containing 3 | Hypo | Predicted to enable guanosine-3',5'-bis(diphosphate) 3'-diphosphatase activity. [provided by Alliance of Genome Resources, Mar 2025] |
| HLA-DMB | 3109 | major histocompatibility complex, class II, DM beta | Hypo | HLA-DMB belongs to the HLA class II beta chain paralogues. This class II molecule is a heterodimer consisting of an alpha (DMA) and a beta (DMB) chain, both anchored in the membrane. It is located in intracellular vesicles. DM plays a central role in the peptide loading of MHC class II molecules by helping to release the CLIP (class II-associated invariant chain peptide) molecule from the peptide binding site. Class II molecules are expressed in antigen presenting cells (APC: B lymphocytes, dendritic cells, macrophages). The beta chain is approximately 26-28 kDa and its gene contains 6 exons. Exon one encodes the leader peptide, exons 2 and 3 encode the two extracellular domains, exon 4 encodes the transmembrane domain and exon 5 encodes the cytoplasmic tail. [provided by RefSeq, Jul 2008] |
| HLA-DPA1 | 3113 | major histocompatibility complex, class II, DP alpha 1 | Hypo | HLA-DPA1 belongs to the HLA class II alpha chain paralogues. This class II molecule is a heterodimer consisting of an alpha (DPA) and a beta (DPB) chain, both anchored in the membrane. It plays a central role in the immune system by presenting peptides derived from extracellular proteins. Class II molecules are expressed in antigen presenting cells (APC: B lymphocytes, dendritic cells, macrophages). The alpha chain is approximately 33-35 kDa and its gene contains 5 exons. Exon one encodes the leader peptide, exons 2 and 3 encode the two extracellular domains, exon 4 encodes the transmembrane domain and the cytoplasmic tail. Within the DP molecule both the alpha chain and the beta chain contain the polymorphisms specifying the peptide binding specificities, resulting in up to 4 different molecules. [provided by RefSeq, Jul 2008] |
| UNC45A | 55898 | unc-45 myosin chaperone A | Hypo | This gene encodes a regulatory component of the progesterone receptor/heat shock protein 90 chaperoning complex, which functions in the assembly and folding of the progesterone receptor. The encoded protein is thought to be essential for normal cell proliferation, and for the accumulation of myosin during development of muscle cells. [provided by RefSeq, Sep 2018] |

**Supplementary Table 4.** Genes overlapping methylated probes and GWAS SNPs

| CHR of Segment with correlated methylated probes identified by SeSAME DMR | Start of Segment with correlated methylated probes identified by SeSAME DMR | End of Segment with correlated methylated probes identified by SeSAME DMR | Segment Pval adjusted BH | Probe ID Illumina Infinium EPICv2 array | Probe CHR | Probe MAPINFO | Beta of lean mass index identified in SeSAME DML | Beta of age identified in SeSAME DML | Pvalue of lean mass index beta identified in SeSAME DML | Pvalue of age beta identified in SeSAME DML | Age significant association with methylation levels | Lean mass index significant association with methylation levels | SNP CHR | SNP rsid | SNP position | SNP reference allele | SNP alternative allele | SNP frequentist add pvalue from GWAS summary stats | Distance between methylated segment and SNP | UCSC RefGene Group from Illumina EPICv2 manifest | UCSC RefGene Name from Illumina EPICv2 manifest |
| --- | --- | --- | --- | --- | --- | --- | --- | --- | --- | --- | --- | --- | --- | --- | --- | --- | --- | --- | --- | --- | --- |
|  |  |  |  |  |  |  |  |  |  |  |  |  |  |  |  |  |  |  |  |  |  |
| **Females** | |  |  |  |  |  |  |  |  |  |  |  |  |  |  |  |  |  |  |  |  |
| chr15 | 90929829 | 90930340 | 1.894E-09 | cg20800831_TC11 | 15 | 90930145 | -0.393481 | 0.0049798 | 0.0064022 | 0.6316665 | FALSE | TRUE | 15 | rs2601189 | 90929874 | A | G | 0.0472293 | 0 | TSS200;3UTR;exon_4;exon_4 | UNC45A;HDDC3;HDDC3;HDDC3 |
| chr6 | 32940689 | 32941125 | 4.773E-05 | cg22324029_BC21 | 6 | 32940689 | -0.268444 | -0.003972 | 0.0426935 | 0.694614 | FALSE | TRUE | 6 | rs544478127 | 32940617 | A | G | 0.0042832 | -72 | NA | NA |
| chr6 | 33008707 | 33009121 | 0.0005426 | cg03780648_TC21 | 6 | 33009049 | -0.583099 | -0.069713 | 0.0127902 | 0.0008974 | TRUE | TRUE | 6 | rs548769287 | 33009678 | T | A | 0.0238597 | 558 | NA | NA |
| chr6 | 33008707 | 33009121 | 0.0005426 | cg27213800_BC21 | 6 | 33009081 | -0.465791 | -0.056892 | 0.0455793 | 0.0053505 | TRUE | TRUE | 6 | rs548769287 | 33009678 | T | A | 0.0238597 | 558 | NA | NA |
| chr6 | 33075797 | 33076569 | 0.0004535 | cg12939283_TC21 | 6 | 33076235 | -0.383949 | -0.017815 | 0.045554 | 0.2354934 | FALSE | TRUE | 6 | rs9394133 | 33079476 | A | C | 0.0301596 | 2908 | NA | NA |
| chr6 | 33072833 | 33073138 | 0.0004003 | cg20617328_TC21 | 6 | 33072833 | -0.413453 | -0.043065 | 0.0449 | 0.0138971 | TRUE | TRUE | 6 | rs9394133 | 33079476 | A | C | 0.0301596 | 6339 | NA | NA |
| chr15 | 90929829 | 90930340 | 1.894E-09 | cg20800831_TC11 | 15 | 90930145 | -0.393481 | 0.0049798 | 0.0064022 | 0.6316665 | FALSE | TRUE | 15 | rs2589944 | 90938720 | C | T | 0.0476121 | 8381 | TSS200;3UTR;exon_4;exon_4 | UNC45A;HDDC3;HDDC3;HDDC3 |
| chr5 | 10250166 | 10251141 | 1.089E-23 | cg11101846_TC21 | 5 | 10250467 | 0.2110382 | 0.0137065 | 0.0065307 | 0.0243321 | TRUE | TRUE | 5 | rs116055676 | 10227350 | T | C | 0.047956 | -22816 | TSS200;TSS200;TSS1500;TSS1500;TSS1500;TSS1500;TSS1500;TSS1500 | CCT5;CCT5;ATPSCKMT;ATPSCKMT;ATPSCKMT;ATPSCKMT;ATPSCKMT;ATPSCKMT |
|  |  |  |  |  |  |  |  |  |  |  |  |  |  |  |  |  |  |  |  |  |  |
| **Males** | |  |  |  |  |  |  |  |  |  |  |  |  |  |  |  |  |  |  |  |  |
| chr15 | 90929829 | 90930340 | 0.0155781 | cg20800831_TC11 | 15 | 90930145 | -0.393481 | 0.0049798 | 0.0064022 | 0.6316665 | FALSE | TRUE | 15 | rs2601189 | 90929874 | A | G | 0.0472293 | 0 | TSS200;3UTR;exon_4;exon_4 | UNC45A;HDDC3;HDDC3;HDDC3 |
| chr6 | 30685630 | 30685773 | 0.0001802 | cg03052182_BC21 | 6 | 30685753 | -0.777911 | -0.073791 | 0.0010514 | 0.00029 | TRUE | TRUE | 6 | rs4713355 | 30685810 | C | T | 0.0191746 | 38 | exon_2;exon_1 | PPP1R18;PPP1R18 |
| chr6 | 30685630 | 30685773 | 0.0001802 | cg12105190_BC21 | 6 | 30685630 | -0.429642 | -0.041469 | 0.0084294 | 0.0027734 | TRUE | TRUE | 6 | rs4713355 | 30685810 | C | T | 0.0191746 | 38 | exon_2;exon_1 | PPP1R18;PPP1R18 |
| chr6 | 30685630 | 30685773 | 0.0001802 | cg12247101_TC11 | 6 | 30685772 | -0.788642 | -0.074496 | 0.0012369 | 0.0003618 | TRUE | TRUE | 6 | rs4713355 | 30685810 | C | T | 0.0191746 | 38 | exon_2;exon_1 | PPP1R18;PPP1R18 |
| chr6 | 30685630 | 30685773 | 0.0001802 | cg25980484_BC21 | 6 | 30685735 | -0.647257 | -0.058284 | 0.0004494 | 0.0001861 | TRUE | TRUE | 6 | rs4713355 | 30685810 | C | T | 0.0191746 | 38 | exon_2;exon_1 | PPP1R18;PPP1R18 |
| chr6 | 25652153 | 25652304 | 0.0265557 | cg04200224_TC11 | 6 | 25652162 | 0.5957759 | 0.045589 | 0.0448655 | 0.0587348 | FALSE | TRUE | 6 | rs72840525 | 25651703 | T | G | 0.0082491 | -450 | TSS200 | SCGN |
| chr6 | 25652153 | 25652304 | 0.0265557 | cg10721149_TC11 | 6 | 25652177 | 0.870018 | 0.0620889 | 0.0107803 | 0.0227955 | TRUE | TRUE | 6 | rs72840525 | 25651703 | T | G | 0.0082491 | -450 | TSS200 | SCGN |
| chr6 | 25652153 | 25652304 | 0.0265557 | cg13586599_BC21 | 6 | 25652155 | 0.473494 | 0.0481205 | 0.0145946 | 0.0037505 | TRUE | TRUE | 6 | rs72840525 | 25651703 | T | G | 0.0082491 | -450 | TSS200 | SCGN |
| chr6 | 25652153 | 25652304 | 0.0265557 | cg13834623_BC11 | 6 | 25652179 | 0.8040873 | 0.0545594 | 0.0480763 | 0.0946867 | FALSE | TRUE | 6 | rs72840525 | 25651703 | T | G | 0.0082491 | -450 | TSS200 | SCGN |
| chr6 | 25652153 | 25652304 | 0.0265557 | cg15562220_BC11 | 6 | 25652167 | 1.0452529 | 0.0729008 | 0.0176896 | 0.0384087 | TRUE | TRUE | 6 | rs72840525 | 25651703 | T | G | 0.0082491 | -450 | TSS200 | SCGN |
| chr17 | 2266208 | 2266696 | 0.014638 | cg02976056_BC21 | 17 | 2266299 | -0.866162 | -0.058056 | 0.0230303 | 0.0558494 | FALSE | TRUE | 17 | rs62067014 | 2265592 | G | A | 0.0440491 | -616 | TSS200 | SMG6 |
| chr17 | 2266208 | 2266696 | 0.014638 | cg07350732_TC21 | 17 | 2266277 | -0.721436 | -0.054029 | 0.0256821 | 0.0387284 | TRUE | TRUE | 17 | rs62067014 | 2265592 | G | A | 0.0440491 | -616 | TSS200 | SMG6 |
| chr17 | 2266208 | 2266696 | 0.014638 | cg09409898_TC21 | 17 | 2266535 | -0.632223 | -0.047728 | 0.0395744 | 0.0646003 | FALSE | TRUE | 17 | rs62067014 | 2265592 | G | A | 0.0440491 | -616 | TSS1500 | SMG6 |
| chr17 | 2266208 | 2266696 | 0.014638 | cg22037995_BC21 | 17 | 2266275 | -0.727133 | -0.055348 | 0.0316458 | 0.0436161 | TRUE | TRUE | 17 | rs62067014 | 2265592 | G | A | 0.0440491 | -616 | TSS200 | SMG6 |
| chr17 | 2266208 | 2266696 | 0.014638 | cg23218897_TC21 | 17 | 2266228 | -0.771577 | -0.061475 | 0.0359803 | 0.0405112 | TRUE | TRUE | 17 | rs62067014 | 2265592 | G | A | 0.0440491 | -616 | TSS200 | SMG6 |
| chr6 | 30329397 | 30329851 | 0.0485544 | cg06230847_BC21 | 6 | 30329817 | 0.6883584 | 0.0358921 | 0.0393066 | 0.1709738 | FALSE | TRUE | 6 | rs3094621 | 30328753 | C | T | 0.048162 | -644 | NA | NA |
| chr6 | 30329397 | 30329851 | 0.0485544 | cg19047804_TC21 | 6 | 30329723 | 0.7828491 | 0.0242704 | 0.0438099 | 0.417603 | FALSE | TRUE | 6 | rs3094621 | 30328753 | C | T | 0.048162 | -644 | exon_3;exon_3;exon_4;exon_3;exon_3;exon_1 | TRIM39;TRIM39;TRIM39;TRIM39;TRIM39;TRIM39-RPP21 |
| chr19 | 8496141 | 8499790 | 0.0155781 | cg01995393_TC21 | 19 | 8498744 | 0.2181111 | 0.0365653 | 0.0331195 | 0.0002317 | TRUE | TRUE | 19 | rs34350946 | 8495115 | T | C | 0.0437469 | -1026 | exon_2 | PRAM1 |
| chr19 | 8496141 | 8499790 | 0.0155781 | cg08474813_TC21 | 19 | 8499789 | 0.5911999 | 0.0554959 | 0.0047847 | 0.0017696 | TRUE | TRUE | 19 | rs34350946 | 8495115 | T | C | 0.0437469 | -1026 | NA | NA |
| chr19 | 8496141 | 8499790 | 0.0155781 | cg14242207_TC21 | 19 | 8499340 | 0.5876588 | 0.0396519 | 0.0002188 | 0.0011969 | TRUE | TRUE | 19 | rs34350946 | 8495115 | T | C | 0.0437469 | -1026 | exon_2 | PRAM1 |
| chr19 | 8496141 | 8499790 | 0.0155781 | cg14324305_TC21 | 19 | 8499717 | 0.5704659 | 0.0429289 | 0.002579 | 0.0046892 | TRUE | TRUE | 19 | rs34350946 | 8495115 | T | C | 0.0437469 | -1026 | exon_2 | PRAM1 |
| chr19 | 8496141 | 8499790 | 0.0155781 | cg23055276_TC21 | 19 | 8499727 | 0.4859929 | 0.0520883 | 0.00027 | 1.734E-05 | TRUE | TRUE | 19 | rs34350946 | 8495115 | T | C | 0.0437469 | -1026 | exon_2 | PRAM1 |
| chr19 | 43983969 | 43984732 | 0.0006053 | cg10590767_TC11 | 19 | 43983975 | -0.165667 | 0.0028882 | 0.0446371 | 0.6474609 | FALSE | TRUE | 19 | rs11665812 | 43986023 | T | C | 0.0352101 | 1292 | TSS1500;TSS1500;TSS1500;TSS1500;TSS1500 | ZNF155;ZNF155;ZNF155;ZNF155;ZNF155 |
| chr19 | 43983969 | 43984732 | 0.0006053 | cg10754510_BC21 | 19 | 43984190 | -0.462298 | 0.0073387 | 9.453E-05 | 0.3231358 | FALSE | TRUE | 19 | rs11665812 | 43986023 | T | C | 0.0352101 | 1292 | 5UTR;exon_1;5UTR;exon_1;5UTR;exon_1;5UTR;exon_1;5UTR;exon_1 | ZNF155;ZNF155;ZNF155;ZNF155;ZNF155;ZNF155;ZNF155;ZNF155;ZNF155;ZNF155 |
| chr19 | 43983969 | 43984732 | 0.0006053 | cg20451226_TC11 | 19 | 43984029 | -0.696711 | 0.007404 | 0.0042056 | 0.6679567 | FALSE | TRUE | 19 | rs11665812 | 43986023 | T | C | 0.0352101 | 1292 | TSS200;TSS200;TSS200;TSS200;TSS200 | ZNF155;ZNF155;ZNF155;ZNF155;ZNF155 |
| chr19 | 43983969 | 43984732 | 0.0006053 | cg23456212_BC11 | 19 | 43984018 | -0.630896 | 0.0047146 | 0.0081382 | 0.7846334 | FALSE | TRUE | 19 | rs11665812 | 43986023 | T | C | 0.0352101 | 1292 | TSS200;TSS200;TSS200;TSS200;TSS200 | ZNF155;ZNF155;ZNF155;ZNF155;ZNF155 |
| chr19 | 43983969 | 43984732 | 0.0006053 | cg24582868_TC21 | 19 | 43984729 | -0.680535 | 0.0059405 | 0.0067325 | 0.7423334 | FALSE | TRUE | 19 | rs11665812 | 43986023 | T | C | 0.0352101 | 1292 | NA | NA |
| chr19 | 43983969 | 43984732 | 0.0006053 | cg24582869_BC21 | 19 | 43984731 | -0.614784 | 0.0088467 | 0.0040861 | 0.5607643 | FALSE | TRUE | 19 | rs11665812 | 43986023 | T | C | 0.0352101 | 1292 | NA | NA |
| chr19 | 43983969 | 43984732 | 0.0006053 | cg27309253_BC21 | 19 | 43984175 | -0.272409 | 0.0003 | 0.0260909 | 0.9739485 | FALSE | TRUE | 19 | rs11665812 | 43986023 | T | C | 0.0352101 | 1292 | TSS200;TSS200;TSS200;TSS200;TSS200 | ZNF155;ZNF155;ZNF155;ZNF155;ZNF155 |
| chr11 | 30017068 | 30017251 | 0.0121439 | cg03506489_BC21 | 11 | 30017241 | 0.5966043 | 0.059536 | 0.01935 | 0.006054 | TRUE | TRUE | 11 | rs142615395 | 30015403 | C | A | 0.0174783 | -1665 | TSS1500 | KCNA4 |
| chr11 | 30017068 | 30017251 | 0.0121439 | cg13161658_BC11 | 11 | 30017250 | 0.8801746 | 0.067548 | 0.0069645 | 0.0103698 | TRUE | TRUE | 11 | rs142615395 | 30015403 | C | A | 0.0174783 | -1665 | TSS1500 | KCNA4 |
| chr11 | 30017068 | 30017251 | 0.0121439 | cg15044957_BC11 | 11 | 30017125 | 1.0329333 | 0.0529963 | 0.0493092 | 0.2000403 | FALSE | TRUE | 11 | rs142615395 | 30015403 | C | A | 0.0174783 | -1665 | TSS200 | KCNA4 |
| chr11 | 30017068 | 30017251 | 0.0121439 | cg15310492_TC11 | 11 | 30017130 | 0.7481949 | 0.0511339 | 0.0396134 | 0.0794684 | FALSE | TRUE | 11 | rs142615395 | 30015403 | C | A | 0.0174783 | -1665 | TSS200 | KCNA4 |
| chr6 | 291687 | 293286 | 1.182E-05 | cg01171360_TC11 | 6 | 293285 | 0.8395309 | -0.010729 | 0.0241162 | 0.7008911 | FALSE | TRUE | 6 | rs3800239 | 295085 | C | A | 0.0322206 | 1800 | NA | NA |
| chr6 | 291687 | 293286 | 1.182E-05 | cg03395511_BC21 | 6 | 291903 | 0.7759308 | -0.027012 | 0.0358047 | 0.342325 | FALSE | TRUE | 6 | rs3800239 | 295085 | C | A | 0.0322206 | 1800 | TSS1500;TSS1500;TSS1500;TSS1500;TSS1500 | DUSP22;DUSP22;DUSP22;DUSP22;DUSP22 |
| chr6 | 291687 | 293286 | 1.182E-05 | cg05064044_BC21 | 6 | 292385 | 0.7488109 | -0.023657 | 0.0296807 | 0.3685077 | FALSE | TRUE | 6 | rs3800239 | 295085 | C | A | 0.0322206 | 1800 | TSS200;TSS200;TSS200;TSS200;TSS200 | DUSP22;DUSP22;DUSP22;DUSP22;DUSP22 |
| chr6 | 291687 | 293286 | 1.182E-05 | cg11235426_TC21 | 6 | 292522 | 0.9421377 | -0.006877 | 0.0006539 | 0.7078938 | FALSE | TRUE | 6 | rs3800239 | 295085 | C | A | 0.0322206 | 1800 | 5UTR;exon_1;5UTR;exon_1;exon_1;exon_1;exon_1 | DUSP22;DUSP22;DUSP22;DUSP22;DUSP22;DUSP22;DUSP22 |
| chr6 | 291687 | 293286 | 1.182E-05 | cg15383120_TC21 | 6 | 291909 | 0.6626811 | -0.014994 | 0.0359323 | 0.5338068 | FALSE | TRUE | 6 | rs3800239 | 295085 | C | A | 0.0322206 | 1800 | TSS1500;TSS1500;TSS1500;TSS1500;TSS1500 | DUSP22;DUSP22;DUSP22;DUSP22;DUSP22 |
| chr6 | 291687 | 293286 | 1.182E-05 | cg17876578_TC21 | 6 | 291859 | 0.7175945 | -0.016792 | 0.0234967 | 0.4821692 | FALSE | TRUE | 6 | rs3800239 | 295085 | C | A | 0.0322206 | 1800 | TSS1500;TSS1500;TSS1500;TSS1500;TSS1500 | DUSP22;DUSP22;DUSP22;DUSP22;DUSP22 |
| chr6 | 291687 | 293286 | 1.182E-05 | cg18110333_BC11 | 6 | 292329 | 1.2036877 | -0.02224 | 0.0148802 | 0.5441044 | FALSE | TRUE | 6 | rs3800239 | 295085 | C | A | 0.0322206 | 1800 | TSS200;TSS200;TSS200;TSS200;TSS200 | DUSP22;DUSP22;DUSP22;DUSP22;DUSP22 |
| chr6 | 291687 | 293286 | 1.182E-05 | cg21548813_BC21 | 6 | 291882 | 0.894914 | -0.025461 | 0.0197124 | 0.3787523 | FALSE | TRUE | 6 | rs3800239 | 295085 | C | A | 0.0322206 | 1800 | TSS1500;TSS1500;TSS1500;TSS1500;TSS1500 | DUSP22;DUSP22;DUSP22;DUSP22;DUSP22 |
| chr6 | 291687 | 293286 | 1.182E-05 | cg26668828_BC11 | 6 | 292823 | 0.8352334 | -0.014075 | 0.041957 | 0.6536913 | FALSE | TRUE | 6 | rs3800239 | 295085 | C | A | 0.0322206 | 1800 | NA | NA |
| chr8 | 95793124 | 95793125 | 0.0081484 | cg21994818_BC21 | 8 | 95793124 | 0.8015525 | 0.0339404 | 0.0002106 | 0.0240043 | TRUE | TRUE | 8 | rs6988572 | 95790833 | G | T | 0.0355674 | -2291 | NA | NA |
| chr8 | 95793124 | 95793125 | 0.0081484 | cg21994818_BC22 | 8 | 95793124 | 0.7569761 | 0.0326424 | 0.0001619 | 0.0190133 | TRUE | TRUE | 8 | rs6988572 | 95790833 | G | T | 0.0355674 | -2291 | NA | NA |
| chr9 | 136843341 | 136843387 | 0.0170593 | cg00755588_TC21 | 9 | 136843341 | -0.524327 | -0.014791 | 0.00091 | 0.1762016 | FALSE | TRUE | 9 | rs72764860 | 136839434 | G | A | 0.0402815 | -3907 | NA | NA |
| chr9 | 136843341 | 136843387 | 0.0170593 | cg14484435_BC21 | 9 | 136843386 | -0.639413 | -0.027313 | 0.0001133 | 0.0164704 | TRUE | TRUE | 9 | rs72764860 | 136839434 | G | A | 0.0402815 | -3907 | NA | NA |
| chr7 | 136115472 | 136115473 | 0.0063462 | cg11756836_BC21 | 7 | 136115472 | -0.764036 | -0.009107 | 0.0001365 | 0.4711746 | FALSE | TRUE | 7 | rs10252342 | 136111305 | A | G | 0.0044599 | -4167 | NA | NA |
| chr7 | 136115472 | 136115473 | 0.0063462 | cg11756836_BC22 | 7 | 136115472 | -0.689085 | -0.009779 | 0.0001765 | 0.4048791 | FALSE | TRUE | 7 | rs10252342 | 136111305 | A | G | 0.0044599 | -4167 | NA | NA |
| chr10 | 103452724 | 103452736 | 0.0474454 | cg09336982_BC11 | 10 | 103452724 | -0.468456 | -0.015658 | 0.0431537 | 0.3813461 | FALSE | TRUE | 10 | rs115878498 | 103447267 | A | G | 0.0408833 | -5457 | TSS1500;TSS1500;TSS1500 | CALHM2;CALHM2;CALHM2 |
| chr10 | 103452724 | 103452736 | 0.0474454 | cg21686808_TC21 | 10 | 103452735 | -0.607076 | -0.008709 | 0.0006058 | 0.4606817 | FALSE | TRUE | 10 | rs115878498 | 103447267 | A | G | 0.0408833 | -5457 | TSS1500;TSS1500;TSS1500 | CALHM2;CALHM2;CALHM2 |
| chr10 | 103452724 | 103452736 | 0.0474454 | cg23175074_TC11 | 10 | 103452733 | -1.183501 | -0.029488 | 0.0018477 | 0.268987 | FALSE | TRUE | 10 | rs115878498 | 103447267 | A | G | 0.0408833 | -5457 | TSS1500;TSS1500;TSS1500 | CALHM2;CALHM2;CALHM2 |
| chr15 | 90929829 | 90930340 | 0.0155781 | cg20800831_TC11 | 15 | 90930145 | -0.393481 | 0.0049798 | 0.0064022 | 0.6316665 | FALSE | TRUE | 15 | rs2589944 | 90938720 | C | T | 0.0476121 | 8381 | TSS200;3UTR;exon_4;exon_4 | UNC45A;HDDC3;HDDC3;HDDC3 |
| chr2 | 43676550 | 43676873 | 0.0024514 | cg08726338_TC21 | 2 | 43676872 | -0.702305 | 0.0041776 | 0.0063089 | 0.8207969 | FALSE | TRUE | 2 | rs6761913 | 43665245 | C | G | 0.044754 | -11305 | TSS1500 | C1GALT1C1L |
| chr2 | 43676550 | 43676873 | 0.0024514 | cg13324389_TC21 | 2 | 43676668 | -0.875289 | 0.0167131 | 0.0134787 | 0.5190151 | FALSE | TRUE | 2 | rs6761913 | 43665245 | C | G | 0.044754 | -11305 | TSS1500 | C1GALT1C1L |
| chr2 | 43676550 | 43676873 | 0.0024514 | cg14454477_TC21 | 2 | 43676761 | -0.942041 | 0.0242086 | 0.0237195 | 0.4418522 | FALSE | TRUE | 2 | rs6761913 | 43665245 | C | G | 0.044754 | -11305 | TSS1500 | C1GALT1C1L |
| chr2 | 43676550 | 43676873 | 0.0024514 | cg18817560_BC21 | 2 | 43676550 | -1.000848 | -0.000156 | 0.0010909 | 0.9939871 | FALSE | TRUE | 2 | rs6761913 | 43665245 | C | G | 0.044754 | -11305 | TSS200 | C1GALT1C1L |
| chr2 | 43676550 | 43676873 | 0.0024514 | cg20563666_BC21 | 2 | 43676703 | -0.836272 | 0.0098156 | 0.0166213 | 0.7046108 | FALSE | TRUE | 2 | rs6761913 | 43665245 | C | G | 0.044754 | -11305 | TSS1500 | C1GALT1C1L |
| chr21 | 44285660 | 44286112 | 5.295E-06 | cg01323542_BC21 | 21 | 44285660 | 0.5687767 | 0.0303096 | 0.0346954 | 0.1513875 | FALSE | TRUE | 21 | rs117813038 | 44298932 | C | A | 0.0074156 | 12821 | TSS1500 | AIRE |
| chr21 | 44285660 | 44286112 | 5.295E-06 | cg08089567_BC11 | 21 | 44286111 | 0.8361857 | 0.0237647 | 0.007804 | 0.3035965 | FALSE | TRUE | 21 | rs117813038 | 44298932 | C | A | 0.0074156 | 12821 | exon_1 | AIRE |
| chr21 | 44285660 | 44286112 | 5.295E-06 | cg08089567_BC12 | 21 | 44286111 | 0.7655138 | 0.021525 | 0.0037403 | 0.2574183 | FALSE | TRUE | 21 | rs117813038 | 44298932 | C | A | 0.0074156 | 12821 | exon_1 | AIRE |
| chr21 | 44285660 | 44286112 | 5.295E-06 | cg11923631_TC11 | 21 | 44285838 | 1.0151504 | 0.0381432 | 0.0200039 | 0.2508428 | FALSE | TRUE | 21 | rs117813038 | 44298932 | C | A | 0.0074156 | 12821 | TSS200 | AIRE |
| chr21 | 44285660 | 44286112 | 5.295E-06 | cg16501323_BC21 | 21 | 44285735 | 0.5949149 | 0.0450177 | 0.0346942 | 0.0485819 | TRUE | TRUE | 21 | rs117813038 | 44298932 | C | A | 0.0074156 | 12821 | TSS200 | AIRE |
| chr21 | 44285660 | 44286112 | 5.295E-06 | cg16717549_TC11 | 21 | 44285816 | 1.0761222 | 0.0406235 | 0.0244512 | 0.2668077 | FALSE | TRUE | 21 | rs117813038 | 44298932 | C | A | 0.0074156 | 12821 | TSS200 | AIRE |
| chr21 | 44285660 | 44286112 | 5.295E-06 | cg17356252_TC21 | 21 | 44285803 | 0.819129 | 0.0321457 | 0.0253357 | 0.252399 | FALSE | TRUE | 21 | rs117813038 | 44298932 | C | A | 0.0074156 | 12821 | TSS200 | AIRE |
| chr21 | 44285660 | 44286112 | 5.295E-06 | cg18689454_BC11 | 21 | 44285811 | 1.1898599 | 0.0458906 | 0.0164115 | 0.2224865 | FALSE | TRUE | 21 | rs117813038 | 44298932 | C | A | 0.0074156 | 12821 | TSS200 | AIRE |
| chr21 | 44285660 | 44286112 | 5.295E-06 | cg18689454_BC12 | 21 | 44285811 | 1.1995287 | 0.0561161 | 0.0154251 | 0.1392693 | FALSE | TRUE | 21 | rs117813038 | 44298932 | C | A | 0.0074156 | 12821 | TSS200 | AIRE |
| chr21 | 44285660 | 44286112 | 5.295E-06 | cg27251412_TC11 | 21 | 44285857 | 1.1165534 | 0.0447471 | 0.0181115 | 0.2137599 | FALSE | TRUE | 21 | rs117813038 | 44298932 | C | A | 0.0074156 | 12821 | TSS200 | AIRE |
| chr3 | 180036745 | 180036828 | 0.0422819 | cg02119363_TC11 | 3 | 180036815 | 1.1589641 | 0.0784323 | 0.004564 | 0.0147877 | TRUE | TRUE | 3 | rs184015719 | 180051441 | C | T | 0.0024542 | 14614 | exon_1;5UTR;exon_1;5UTR;exon_1;5UTR;exon_1;5UTR;exon_1;5UTR;exon_1;5UTR;exon_1;5UTR;5UTR;exon_1;5UTR;exon_1;5UTR;exon_1;exon_1;5UTR | PEX5L;PEX5L;PEX5L;PEX5L;PEX5L;PEX5L;PEX5L;PEX5L;PEX5L;PEX5L;PEX5L;PEX5L;PEX5L;PEX5L;PEX5L;PEX5L;PEX5L;PEX5L;PEX5L;PEX5L;PEX5L;PEX5L |
| chr3 | 180036745 | 180036828 | 0.0422819 | cg04894619_BC11 | 3 | 180036827 | 1.1805016 | 0.0788826 | 0.0044323 | 0.0154893 | TRUE | TRUE | 3 | rs184015719 | 180051441 | C | T | 0.0024542 | 14614 | exon_1;5UTR;exon_1;5UTR;exon_1;5UTR;exon_1;5UTR;exon_1;5UTR;exon_1;5UTR;exon_1;5UTR;5UTR;exon_1;5UTR;exon_1;5UTR;exon_1;exon_1;5UTR | PEX5L;PEX5L;PEX5L;PEX5L;PEX5L;PEX5L;PEX5L;PEX5L;PEX5L;PEX5L;PEX5L;PEX5L;PEX5L;PEX5L;PEX5L;PEX5L;PEX5L;PEX5L;PEX5L;PEX5L;PEX5L;PEX5L |
| chr3 | 180036745 | 180036828 | 0.0422819 | cg13473356_TC11 | 3 | 180036825 | 0.9265562 | 0.0814615 | 0.0227987 | 0.0156794 | TRUE | TRUE | 3 | rs184015719 | 180051441 | C | T | 0.0024542 | 14614 | exon_1;5UTR;exon_1;5UTR;exon_1;5UTR;exon_1;5UTR;exon_1;5UTR;exon_1;5UTR;exon_1;5UTR;5UTR;exon_1;5UTR;exon_1;5UTR;exon_1;exon_1;5UTR | PEX5L;PEX5L;PEX5L;PEX5L;PEX5L;PEX5L;PEX5L;PEX5L;PEX5L;PEX5L;PEX5L;PEX5L;PEX5L;PEX5L;PEX5L;PEX5L;PEX5L;PEX5L;PEX5L;PEX5L;PEX5L;PEX5L |
| chr3 | 180036745 | 180036828 | 0.0422819 | cg23346462_TC11 | 3 | 180036745 | 0.6103531 | 0.0313844 | 0.0279668 | 0.1472876 | FALSE | TRUE | 3 | rs184015719 | 180051441 | C | T | 0.0024542 | 14614 | exon_1;5UTR;exon_1;5UTR;exon_1;5UTR;exon_1;5UTR;exon_1;5UTR;exon_1;5UTR;exon_1;5UTR;5UTR;exon_1;5UTR;exon_1;5UTR;exon_1;exon_1;5UTR | PEX5L;PEX5L;PEX5L;PEX5L;PEX5L;PEX5L;PEX5L;PEX5L;PEX5L;PEX5L;PEX5L;PEX5L;PEX5L;PEX5L;PEX5L;PEX5L;PEX5L;PEX5L;PEX5L;PEX5L;PEX5L;PEX5L |
| chr16 | 57798088 | 57798319 | 5.934E-06 | cg00947878_BC11 | 16 | 57798093 | 1.0920219 | 0.0829863 | 0.0004453 | 0.0008486 | TRUE | TRUE | 16 | rs534752446 | 57814558 | C | G | 0.0321751 | 16240 | exon_2;exon_2;exon_2;TSS200;TSS200;TSS200 | KIFC3;KIFC3;KIFC3;KIFC3;KIFC3;KIFC3 |
| chr16 | 57798088 | 57798319 | 5.934E-06 | cg13352096_TC11 | 16 | 57798173 | 1.2030905 | 0.0810442 | 0.0012663 | 0.0053193 | TRUE | TRUE | 16 | rs534752446 | 57814558 | C | G | 0.0321751 | 16240 | exon_2;exon_2;exon_2;TSS200;TSS200;TSS200 | KIFC3;KIFC3;KIFC3;KIFC3;KIFC3;KIFC3 |
| chr16 | 57798088 | 57798319 | 5.934E-06 | cg13750905_TC21 | 16 | 57798088 | 0.6496272 | 0.0461296 | 0.0016939 | 0.0047761 | TRUE | TRUE | 16 | rs534752446 | 57814558 | C | G | 0.0321751 | 16240 | exon_2;exon_2;exon_2;TSS200;TSS200;TSS200 | KIFC3;KIFC3;KIFC3;KIFC3;KIFC3;KIFC3 |
| chr16 | 57798088 | 57798319 | 5.934E-06 | cg15627357_TC11 | 16 | 57798143 | 0.9360565 | 0.0755268 | 0.0066143 | 0.0072553 | TRUE | TRUE | 16 | rs534752446 | 57814558 | C | G | 0.0321751 | 16240 | exon_2;exon_2;exon_2;TSS200;TSS200;TSS200 | KIFC3;KIFC3;KIFC3;KIFC3;KIFC3;KIFC3 |
| chr16 | 57798088 | 57798319 | 5.934E-06 | cg26927427_TC21 | 16 | 57798318 | 0.5929229 | 0.0271035 | 0.0018948 | 0.0531414 | FALSE | TRUE | 16 | rs534752446 | 57814558 | C | G | 0.0321751 | 16240 | TSS1500;TSS1500;TSS1500 | KIFC3;KIFC3;KIFC3 |
| chr19 | 45234345 | 45234535 | 0.0063462 | cg00449767_TC11 | 19 | 45234345 | 1.2273762 | 0.0455838 | 0.0048727 | 0.1552642 | FALSE | TRUE | 19 | rs79745266 | 45215805 | C | T | 0.0243768 | -18540 | exon_3 | EXOC3L2 |
| chr19 | 45234345 | 45234535 | 0.0063462 | cg01565314_BC11 | 19 | 45234352 | 1.4785064 | 0.0499591 | 0.0035779 | 0.1749984 | FALSE | TRUE | 19 | rs79745266 | 45215805 | C | T | 0.0243768 | -18540 | exon_3 | EXOC3L2 |
| chr19 | 45234345 | 45234535 | 0.0063462 | cg08882547_TC11 | 19 | 45234534 | 0.9334999 | 0.0965711 | 0.0054296 | 0.0009428 | TRUE | TRUE | 19 | rs79745266 | 45215805 | C | T | 0.0243768 | -18540 | exon_3 | EXOC3L2 |
| chr19 | 45234345 | 45234535 | 0.0063462 | cg09450024_BC11 | 19 | 45234365 | 1.4876127 | 0.0565019 | 0.0081376 | 0.1777388 | FALSE | TRUE | 19 | rs79745266 | 45215805 | C | T | 0.0243768 | -18540 | exon_3 | EXOC3L2 |
